# Strength performance of low-bearing-capacity clayey soils stabilized with ladle furnace slag

**DOI:** 10.1007/s11356-023-29375-y

**Published:** 2023-08-30

**Authors:** Ana B. Espinosa, Víctor Revilla-Cuesta, Marta Skaf, Roberto Serrano-López, Vanesa Ortega-López

**Affiliations:** 1 Department of Construction, Escuela Politécnica Superior, University of Burgos, c/ Villadiego s/n, 09001 Burgos, Spain; 2 Department of Civil Engineering, Escuela Politécnica Superior, University of Burgos, c/ Villadiego s/n, 09001 Burgos, Spain; 3 Universidad de Burgos Escuela Politecnica Superior, c/ Villadiego s/n, Burgos, 09001 Spain

**Keywords:** Soil stabilization, Ladle furnace slag, Clayey soil, Bearing capacity, Recycling, Road construction

## Abstract

In this paper, the performance of ladle furnace slag (LFS), a by-product of secondary steel refining, is evaluated as a binder to stabilize clayey soils of low bearing capacity. The aim is to define whether additions of this by-product to clayey soil can stabilize the soil in accordance with the technical specifications of Spanish standards. To do so, three different soils stabilized with 5% LFS were compared with the same soils stabilized with 2% lime and with no stabilization, in order to investigate their different behaviors. The chemical and mineralogical characterizations of all the soil mixes were conducted using X-ray fluorescence, X-ray diffraction, and scanning electron microscopy. The Atterberg limit test was used to study the plastic behavior of the soils, and the results of compaction, bearing capacity, unconfined compressive strength, and direct shear strength (cohesion and friction angle) tests defined their strength characteristics. The analysis was completed with the pH monitoring of the mixes along the curing time in order to relate the pH changes with the strength evolution. The addition of LFS to the soils has resulted in an increase in the liquid limit and plastic limit, causing therefore a slight decrease in the plasticity index. All the soils showed increases between 30% and 70% in their California Bearing Ratios immediately after mixing with 5% LFS, and after 90 days of curing, improvements of 30–188% in their unconfined compressive strength were noted in comparison with untreated soil, which were higher than the lime-stabilized soils. The cohesion of soils stabilized with LFS at 28 days of curing obtained improvements ranging from 40 to 300% depending on the type of soil. However, the friction angle showed a slight increase of 10% in two of the soils and zero in another. The high initial pH in LFS-stabilized soils was maintained during the curing time, which favored the development of pozzolanic reactions that improve the soil strength. These results confirmed that the substitution of lime with LFS is a feasible option for soil stabilization.

## Introduction

The siderurgical industry is among the most important global economic activities. In 2022, world crude steel production amounted to 1831.5 million tons (Mt) (World Steel Association 2023). The main waste generated during iron and steel production is slag, formed from metallic oxides that rise to the surface of the molten metal as it cools and solidifies (Yildirim and Prezzi 2011). In 2022, global production of slag, estimated at around 10–15% of crude steel production, was within the region of 190 Mt and 290 Mt (USGS 2023).

Steel-production processes differ from country to country, but can currently be divided, in a simplified way, into two types. On the one hand, there is an integral iron and steel industry, in which iron ore is converted into a raw material in a blast furnace (BF), which is followed by a decarburization process, generally in blown oxygen converters (basic oxygen furnace-BOF). On the other hand, scrap iron is usually melted in electric arc furnaces (EAF) in an electric cycle. After this “primary metallurgy,” a second refining process is required to obtain high-quality steel that consists of subjecting the liquid steel to a dephosphorization process in an EAF or BOF, which is subsequently poured into a ladle where it is deoxidized, desulfurized, and alloyed under the protection of a basic slag (Setién et al. 2009). Then, the slag is poured from the ladle in a liquid state, cooled to ambient temperature, and piled up in large slag heaps outside. This steelmaking by-product is ladle furnace slag (LFS).

Utilizing these by-products for new applications, such as construction (Akinmusuru 1991; Geiseler 1996; Motz and Geiseler 2001) and the agriculture sector (Proctor et al. 2000), is a sensible approach that aligns with the principles of the circular economy, effectively mitigating the environmental impact arising from the significant volumes of slag generated in iron and steel processes. Previous experiences have explored the reuse of certain by-products from the steel industry, mainly at an experimental level. The primary focus has been on reusing blast furnace slag (BFS) (Wild et al. 1998), electric arc furnace slag (EAFS) (Yildirim and Prezzi 2017), and converter slag or basic oxygen furnace slag (BOFS) (Kambole et al. 2017). However, research into the reuse of ladle furnace slag (LFS) is less widespread (Serjun et al. 2013; Santamaría et al. 2021). Although the global annual LFS production is set at about 30 Mt per year (Mahoutian and Shao 2016), this type of slag is usually deposited in landfills due to the wide variability in its chemical composition and crystalline structure depending on its origin (Setién et al. 2009), which complicates its feasibility.

Over recent years, greater ecological awareness in all of its social dimensions, the need to reduce the use of natural resources, and the economic opportunity of reusing potentially useful co-products for other industrial materials have prompted several investigations aimed at finding feasible and economically profitable applications of LFS, such as aggregate in the manufacture of structural mortars (Adolfsson et al. 2011; Santamaría et al. 2016; Rosales et al. 2017), masonry mortars (Rodriguez et al. 2009; Manso et al. 2011), vibrated concrete (Papayianni and Anastasiou 2010; Polanco et al. 2011) and self-compacting concrete (Anastasiou et al. 2014), cement production (Richardson and Cabrera 2000; Akin Altun and Yilmaz 2002), soil stabilization (Kanagawa and Kuwayama 1997; Montenegro et al. 2013), and environmental engineering (Herrmann et al. 2010; Radenović et al. 2013) and granular material for backfills (Maghool et al. 2017a).

The stabilization of low-quality clayey soils is a crucial civil engineering practice enhancing the performance of road infrastructures, foundations, and slope stability. This process aims to improve various aspects, such as strength, stress-strain properties, volumetric stability, permeability, and durability (Bell and Coulthard 1990). These improvements can be achieved through a range of methods, including mechanical, biological, physical, chemical, or thermal procedures (Ortega-López et al. 2017). Among the diverse stabilization techniques available, chemical stabilization stands out as the most widely used method in the geotechnical sector, primarily due to its cost-effectiveness (Katz et al. 2001). Traditionally, chemical stabilization has relied on additives like cement (Consoli et al. 2007), lime (Bell 1996; Boardman et al. 2001; Dash and Hussain 2012; Ghobadi et al. 2014), and fly ash (Kim et al. 2019; Manh Do et al. 2019). Non-traditional agents encompass additional additives that undergo chemical reactions with the soil and/or other additives. These materials include industrial by-product materials, other waste products with calcium oxide content, such as waste paper sludge ash, sulphonated oils, ionic compounds, and polymers (Petry and Little 2002; Ikeagwuani and Nwonu 2019).

By incorporating industrial by-products, economic benefits are provided, and they also contribute to a more sustainable approach to soil stabilization, effectively reducing the carbon footprint (Behnood 2018; Xu and Yi 2019). Therefore, numerous new research challenges have been undertaken to develop environmentally friendly stabilizers based on these innovative materials (Nidzam and Kinuthia 2010; Wilkinson et al. 2010a, b, James and Pandian 2016; Brand and Fanijo 2020), such as ground granulated blast furnace slag (GGBFS) (Obuzor et al. 2012; Hossein Rafiean et al. 2020; Seco et al. 2021), basic oxygen furnace slag (BOFS) (Poh et al. 2006; Mahieux et al. 2009; Kambole et al. 2017), button fly ash (Yoon et al. 2009), and cement kiln dust (Mosa et al. 2017).

Several studies have highlighted the potential of LFS in improving problematic soils, enhancing their plasticity, strength, and drainage properties (Akinwumi 2014; Montenegro-Cooper et al. 2019). Understanding the complete range of chemical, mineralogical, and morphological properties of slags is crucial, as their mechanical and cementitious characteristics play a vital role in successful soil stabilization (Manso et al. 2005; Setién et al. 2009; Yildirim and Prezzi 2011; Marinho et al. 2017). Considering the composition of LFS and its pozzolanic properties, which are similar to lime, several research groups have explored the substitution of lime with LFS (Manso et al. 2013; Montenegro et al. 2013; Ortega-López et al. 2017; Brand et al. 2020) and their combined use (Shen et al. 2019; Xu and Yi 2019). The primary constituents of LFS are calcium oxide and magnesium oxide, both being of a basic nature and accounting for over 60% of the total weight. Other components, comprising around 30% of the weight, are oxides of an acidic nature such as silica and aluminum oxide. Additionally, LFS contains various other components in smaller proportions (less than 10% in weight), including calcium fluoride, sulfide, and oxides of iron, manganese, titanium, and alkaline elements (Setién et al. 2009).

The expansive properties of LFS cause great limitations to its direct use as a granular material for civil engineering applications (Papayianni and Anastasiou 2006; Setién et al. 2009; Maghool et al. 2017b), so many researchers have therefore analyzed the unstable character of this material (Wang et al. 2010; Montenegro-Cooper et al. 2019). The hydration and carbonation of the free-CaO (lime) and free-MgO (periclase) contained in LFS provoke the appearance of portlandite (Ca(OH)_2_), calcite (CaCO_3_), brucite (Mg(OH)_2_), and magnesite (MgCO_3_), among others. These compounds are associated with significant volumetric expansion, especially remarkable for MgO reactions, whose swelling can in the long term reach values of up to 40% or more (Ortega-López et al. 2014). These possible expansive reactions must therefore be considered in the applications of this industrial by-product.

In this paper, an experimental program designed to evaluate the applicability of LFS in the stabilization of low-bearing-capacity clayey soils is presented. Three soils of different origin and composition were selected, all of them classified as unsuitable for their direct use as filler material in civil works. A fixed content of 5% LFS was established, based on previous studies of the authors (Manso et al. 2013; Ortega-López et al. 2014). Moreover, since soil stabilization with lime has been one of the oldest and most widespread techniques, it was decided to prepare control samples of each soil with a lime percentage of 2%. The main research objective of this study was to verify that the use of LFS in low-bearing-capacity soil stabilization yielded satisfactory geotechnical results, comparable to conventional stabilization results with lime, with the subsequent environmental benefit. To do so, the tests defined in current Spanish standards (Ministerio de Fomento 2015) were performed on all the mixes to corroborate their behavior, focusing on their mechanical properties and volumetric stability and comparing the experimental soil-LFS results with those of control soil-lime mixes. Hence, an exhaustive test program was developed, in which the plasticity, compaction, bearing capacity, unconfined compressive strength, and volumetric stability of the mixes were analyzed. Moreover, another contribution of this research was the analysis and evolution of the shear strength parameters determined with the direct shear test, in order to obtain the necessary analytical values required for the bearing capacity calculation based on traditional geotechnical formulas (Das 1999) or using finite element software. The evolution of the pH over the curing time was analyzed in order to correlate its variation with the strength development. Furthermore, a detailed study of chemical, mineralogical, and microstructural characterization of the stabilized soils was conducted with advanced techniques such as X-ray fluorescence (XRF), X-ray diffraction (XRD), and scanning electron microscopy (SEM), in order to ascertain the main changes that lead to improved soil performance after mixing the soils with LFS. In this research, it was found that small percentages of LFS stabilizer generated improvements in the plastic and strength behavior of the treated soil.

## Materials and methodology

### Raw materials

#### Natural soils

Three clayey soils of low bearing capacity were selected that were not acceptable in terms of the Spanish technical specifications (PG-3) (Ministerio de Fomento 2002) on the use of soil in civil works such as road embankments, bases, and subbases. The samples were collected during earthworks at three different locations in the province of Burgos (Spain), identified as SA (Soil Argaño), SB (Soil Bakimet), and SV (Soil Villalonquejar), according to their origin. Their real final destinations all differed: SA was reused as embankment filler following stabilization with lime; SB was used as filler within a landscaped area; and SV was removed to a temporary storage area for future applications.

In Article 512 of the Spanish regulation PG-3 (Ministerio de Fomento 2015), two categories of lime-stabilized soils are defined: soil type 1, where the California Bearing Ratio (CBR) of the mix at 7 days is no less than 6 and the lime content must be under 2%; and soil type 2, where the CBR of the mix at 7 days is no less than 12 and the lime content must remain above 3%. In addition, this regulation establishes that natural soils can be stabilized in situ with lime, if they satisfy several specifications:

- Regarding its granulometry, 100% should pass through an 80-mm sieve and more than 15% through a 0.063-mm sieve.
- Concerning its chemical composition, the organic-matter content must not exceed 2% to obtain a type 1 stabilized soil and 1% for a type 2 stabilized soil.
- In both soil types, the soluble-sulfate content should be less than 0.7%.
- With respect to plasticity, soils must have a plasticity index (PI) higher than 12% for a type 1 stabilized soil and between 12 and 40% for a type 2 stabilized soil. If the value of 40% is exceeded, the soil must be mixed with lime in two stages.

Table 1 shows a summary of the main physical and chemical soil properties, together with their classification according to Unified Soil Classification System (USCS) and Spanish classification regulation (Ministerio de Fomento 2002). Following this characterization, three soils were suitable for stabilization according to the PG-3 Spanish regulation (Ministerio de Fomento 2015). Although the SB soil did not fulfill the plasticity requirement, it met the rest of the criteria, so it was also considered appropriate for its experimental stabilization. According to the Spanish classification, all the clays were marginal soils. According to USCS, the SA and SV soils were classified as CL, indicating that they were inorganic clays of medium plasticity, and the SB soil was classified as SC-SM, indicating silty-clay sands with a high percentage of fine material.

**Table 1:** Properties and classification of soils

Properties		SA	SB	SV
Particle size distribution ISO 17892-4:2016	% passing # 80 mm	100	100	100.0
	% passing # 5 mm	97.7	91.7	89.7
	% passing # 0.08 mm	79.3	45.8	67.7
Specific gravity of particles (Mg/m^3^) ISO 17892-3:2015		2.69	2.69	2.72
Organic matter content (%) UNE103 204:2019		0.96	0.89	0.94
Soluble sulfates (% SO_3_) UNE 103 205:2019		0.02	0.16	0.06
LL (%) ISO 17892-12:2012		31.00	21.20	46.40
PL (%) ISO 17892-12:2012		16.10	16.20	23.30
PI (%)		15.00	5.00	23.10
USCS classification*		CL	SC-SM	CL
Spanish classification		Marginal	Marginal	Marginal

*CL, clays; *SC*, clayey sands; *SM*, silty sands

Apart from the physical properties of the soils, a series of tests were performed to determine their chemical, mineralogical, and micro-texture characteristics through X-ray fluorescence (XRF), X-ray diffraction (XRD), and scanning electron microscopy (SEM).

**Fig. 1:**
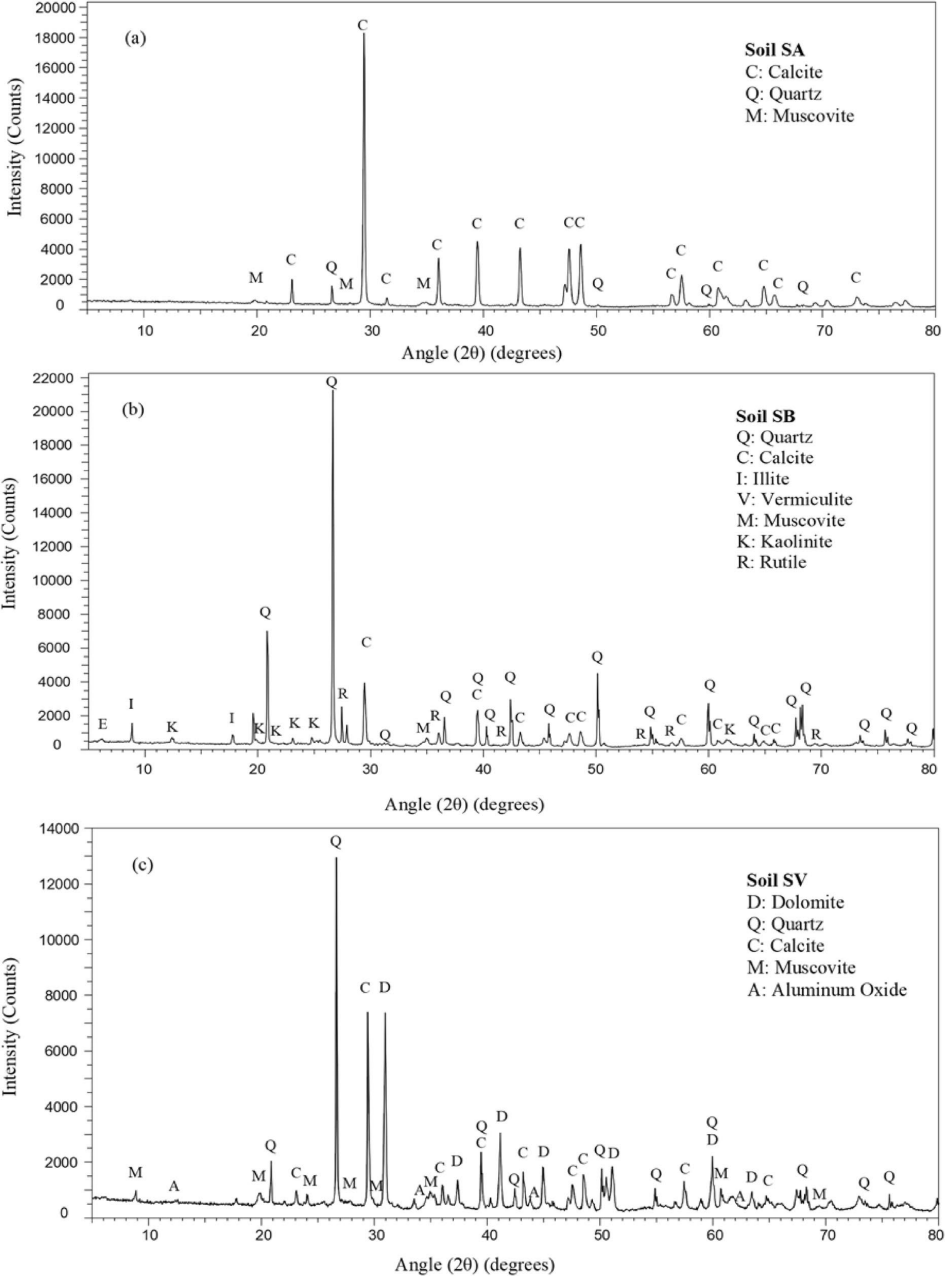
XRD diffraction pattern of natural soils: **a** SA; **b** SB; **c** SV

**Fig. 2:**
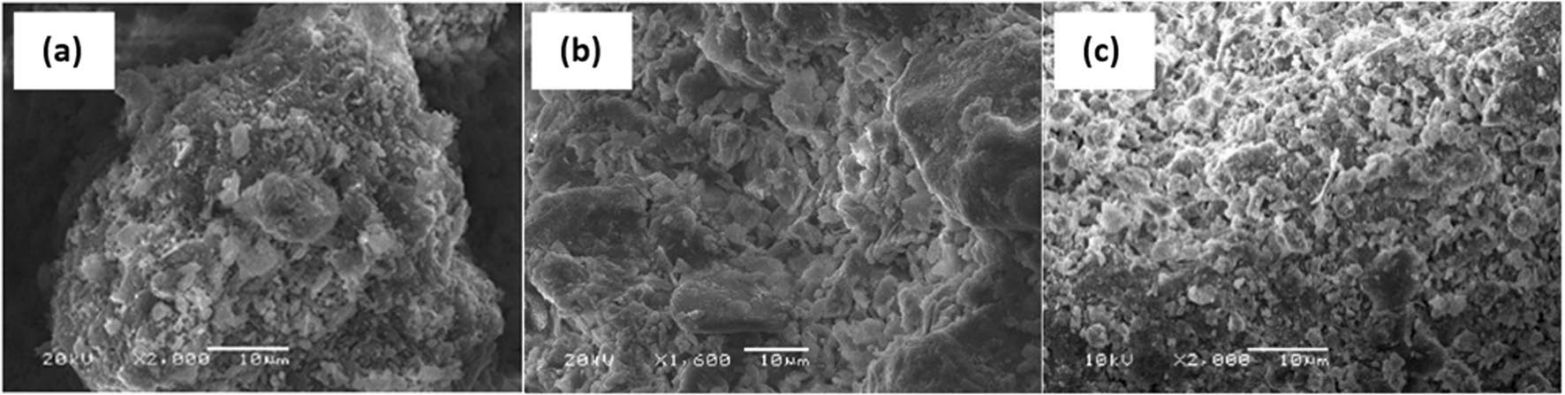
SEM images of natural soils: **a** SA; **b** SB; **c** SV, at different magnifications (scale bar: 10 μm)

- A Thermo Electron Corporation, ARL ADVAT XP Sequential X-Ray Fluorescence spectrometer was used for the chemical evaluation of the soils. The UNIQUANT 5.47 program was used to determine the concentration of the oxides, and the results were expressed in % (limit of detection 10 ppm). Table 2 contains the results obtained in the XRF test for the natural soils under study.
- A Bruker D8 Discover Davinci X-Ray Diffractometer was used for the mineralogical characterization of the soils. The equipment had the following technical specifications: LynxEye XE_T detector, Cu radiation, voltage 40 kV, intensity 30 mA, scanning range 5° 2*θ*–80° 2*θ*, step size of 0.05° 2*θ*, and time per step 1 s. The main mineralogical components and their concentration in the three natural soils are shown in Table 3 and Fig. 1, which contains their diffraction patterns.
- A JEOL JSM-6460LV Scanning Electron Microscope (SEM) with backscattered electron, secondary electron, and X-ray energy dispersive detectors was used to perform the SEM analysis for the morphological characterization of the materials. The SEM equipped with a high vacuum system produces images at a magnification power of times 300,000 and analysis of elemental chemical composition. Figure 2 shows SEM images of the three soils.

**Table 2:** XRF results of natural soils and binders (% by weight)

Weight (W %)	SA (W %)	SB (W %)	SV (W %)	LFS (W %)	Lime (W %)
CaO	59.58	12.83	26.82	56.7	92.3
SiO_2_	20.52	69.06	45.25	17.7	1.20
MgO	1.93	1.16	8.25	9.60	1.40
Fe_2_O_3_	3.15	3.26	3.51	2.20	0.30
Al_2_O_3_	8.27	11.41	10.85	6.60	2.10
SO_3_	0.29	0.08	0.25	0.92	0.10
TiO_2_	0.25	0.63	0.38	0.34	0.80
K_2_O+Na_2_O	1.68	2.59	2.55	0.10	0.90
F	1.89	N.D.	0.15	N.D.	N.D.
LOI 110*	2.18	1.72	2.84	-	-
LOI 550*	5.64	3.99	6.62	-	-

*LOI, loss on Ignition 110 °C/550 °C, calculated from the mass loss obtained in ignition analysis, mainly corresponding to H_2_O and CO_2_

**Table 3:** Main mineralogical phases of natural soils obtained through XRD

Mineral	Chemical formula	Concentration level		
		SA	SB	SV
Quartz	SiO_2_	Major	Major	Major
Calcite	CaCO_3_	Major	Major	Major
Muscovite	KAl_2_(AlSi_3_O_10_)(OH)_2_	Major	Major	Major
Illite-2M2	(K,H_3_0)Al_2_(Si_3_Al)O_10_(OH)2·H_2_O	-	Major	-
Vermiculite-2M	Mg3.41Si2.86Al1.14O_10_(OH)_2_(H_2_O)3.72	-	Trace	-
Kaolinite	Al_2_(Si_2_O_5_)(OH)_4_	-	Major	-
Rutile	TiO_2_	-	Minor	-
Dolomite	CaMg(CO_3_)_2_	-	-	Major
Aluminum Oxide	Al_2_O_3_	-	-	Minor

All the three soils had quartz, calcite, and muscovite in their composition. In fact, the SA soil was only composed of those three minerals. The SB soil also had illite and kaolinite, small concentrations of rutile, and traces of vermiculite. In contrast, the SV soil presented, in addition to quartz, calcite, and muscovite, dolomite, and small concentrations of aluminum oxide. SEM images revealed the discontinuous structure of the three soils, with smooth morphologies, dispersed small-sized particles, where the voids between them could be appreciated.

#### Lime

The hydrated lime CL90-S, identified as C, was manufactured by Cementos Tudela Veguin (Oviedo, Spain). The chemical composition of this material is shown in Table 2.

#### Ladle furnace slag

**Fig. 3:**
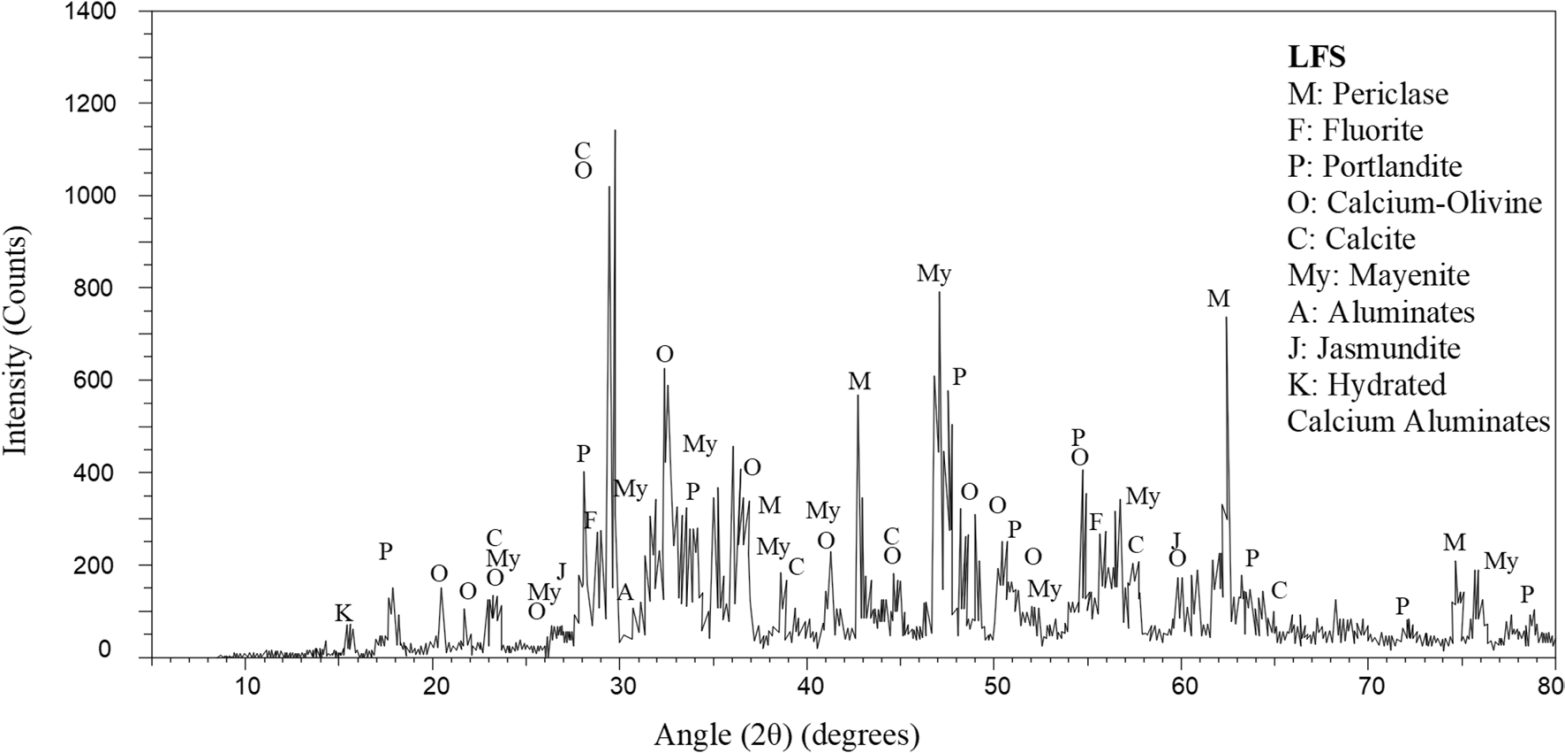
XRD diffraction pattern of LFS

The ladle furnace slag (LFS) used in this research (labelled E) was a by-product obtained from the metallurgical activity at Acería Tubos Reunidos (Amurrio, Spain). It was a powdery material whose composition, similar to cement and/or limes, contained calcium and magnesium silicates and aluminates, and it was therefore expected to be suitable for use in soil stabilization (Ortega-López et al. 2017). The LFS fraction used was smaller than 1 mm in size. Table 2 contains the chemical composition of the LFS, determined by XRF, and Table 4 contains its main mineralogical phases obtained by XRD (Fig. 3), using the same procedures and equipment as for the natural soils.

**Table 4:** Main mineralogical phases of LFS

Mineral	Chemical formula	Concentration level
Periclase	MgO	Medium
Fluorite	CaF_2_	Minor
Portlandite	Ca(OH)_2_	Medium
Calcium–olivine	Ca_2_SiO_4_	Major
Calcite	CaCO_3_	Medium
Mayenite	Ca_12_Al_14_O_33_	Medium
Aluminates	A_3_C_5_	Traces
Jasmundite	Ca_11_(SiO_4_)_4_O_2_S	Minor
Hydrated calcium aluminates	Ca_3_Al_2_[(OH)_4_]	Traces

The hydraulic index (*i*) can be determined on the basis of the LFS chemical composition obtained by XRD. The index is defined as a ratio between the acidic and the basic oxides that compose a material (Eq. (1)). The slag in use yielded a hydraulic index of 0.4, a typical value for a hydraulic lime, so it was classified as standard hydraulic (hydraulic indexes: air lime 0–0.1; weakly hydraulic 0.1–0.16; averagely hydraulic 0.16–0.31; standard hydraulic 0.31–0.42; strongly hydraulic 0.42–0.5) (Coutinho and Gonçalves 1988; Marinho et al. 2017).

1 $$i=\frac{\%{\textrm{SiO}}_2+\%{\textrm{Fe}}_2{\textrm{O}}_3+\%{\textrm{Al}}_2{\textrm{O}}_3}{\%\textrm{CaO}+\%\textrm{MgO}}$$

The presence of reactive SiO_2_ and Ca(OH)_2_ in LFS implies that it can show self-cementing properties and pozzolanic properties as a binder (Malhotra and Mehta 2004), as several researchers have confirmed (Shi 2002, 2004; Adolfsson et al. 2011; Belhadj et al. 2012; Papayianni and Anastasiou 2012). The cementitious capacity of slag is defined by its alkaline character, according to the ratio (CaO+MgO)/(SiO_2_+Al_2_O_3_) (CEDEX 2013). Taylor (1997) indicated that steel slags must have a basic character greater than 1.8 points to be used as cementitious materials. The alkalinity of the LFS, as per its bulk composition in Table 2, was 2.73, which is indicative of high reactivity (Shi and Qian 2000), so it can be considered as a low-strength Portland cement clinker (Shi and Day 1999).

Finally, hydration and carbonation of free lime (CaO) and magnesia (MgO) are the main causes of the volumetric expansion of LFS that is also the main drawback for its use in civil-engineering applications (Motz and Geiseler 2001). Exposure to natural weathering is an efficient technique to minimize the deferred formation of expansive oxides that can cause problems in the long term when using LFS (Diniz et al. 2017). A problem that can be minimized when most of the free lime in the LFS has already been partially hydrated, in the form of portlandite, Ca(OH)_2_, and even slightly carbonated, in the form of calcite, CaCO_3_. However, although magnesium oxides were present in lower concentrations than calcium oxides, they led to slow carbo-hydration reactions, which resulted in their expansion in the medium to long term. Such behavior must therefore be controlled during application (Manso et al. 2013).

### Design of soil-stabilized mixes

The three natural soils were mixed with 5% LFS, in order to evaluate the potential use of LFS as a stabilizing agent, considering previous studies of this research group (Manso et al. 2013; Ortega-López et al. 2014). As a reference mix, the soils were also mixed with 2% lime, which is the minimum binder content according to the standards (Ministerio de Fomento 2015), aiming to reduce the consumption of raw materials that are also non-sustainable. Previous research, such as studies conducted by Eades and Grim (1966), Bell (1996), Dash and Hussain (2012) and Ghobadi et al. (2014), indicated that stabilizations with low percentages of lime (1–3%) also lead to significant strength improvements over the curing time. In that way, it was expected to compare both types of stabilizations with a conventional binder (lime) and with an alternative binder (LFS), with the purpose of obtaining mixes of similar quality to be used in road sub-bases. Furthermore, the performance of the non-stabilized soils was also evaluated.

The identification of the mixes in the study were as follows:

- Soil SA mixed with 2% lime (SAC2).
- Soil SA mixed with 5% LFS (SAE5).
- Soil SB mixed with 2% lime (SBC2).
- Soil SB mixed with 5% LFS (SBE5).
- Soil SV mixed with 2% lime (SVC2).
- Soil SV mixed with 5% LFS (SVE5).

### Experimental methodology

A series of physical and chemical characterization tests were performed on the 9 soil samples (3 natural soils, 3 soil-lime mixes, 3 soil-LFS mixes) as per Spanish standards (Ministerio de Fomento 2002). The following tests were conducted: plasticity, modified compaction, bearing capacity, unconfined compressive strength, direct shear strength, volume stability, and pH evolution Euronorm (n.d.), the experimental processes of which are detailed below. In addition, XRD and SEM analyses were also conducted on the stabilized mixes after curing for 90 days following the same procedures and using the same equipment as in the raw-material analysis. Most of the chemical reactions were thought to occur during the medium-term.

#### Atterberg limits

Atterberg limit tests were performed to determine both the liquid and the plastic limit and the plasticity index, according to ISO 17892-12 (ISO 2012). These tests were performed immediately after mixing with the established binder percentages, with no time to start complex chemical processes, in order to verify the effects of the binders on the plasticity of the mixes.

2.3.2. Modified compaction test

The Optimum Moisture Content (OMC) and Maximum Dry Density (MDD) were determined for each soil sample, which were the reference values for further determinations and tests. The Modified Proctor (MP) test was performed, according to ASTM D1557-12 (2012), whose compaction energy was 2,632 J/cm^3^. A minimum of 5 points was obtained to define the compaction curves.

#### Unconfined compressive strength test

Unconfined compressive strength (UCS) is one of the most common tests to estimate the stiffness of materials used in pavements. In fact, it is an index value that determines the applicability of a material in the different areas of a pavement structure. This test consists of applying an axial vertical load on a cylindrical specimen without lateral confinement, controlling the stress-strain conditions, according to EN ISO 17892-7 (ISO 2017). UCS is defined as the maximum unit strength supported by the specimen in the loading process at a constant speed. The cylindrical test specimens were prepared in a Harvard mold (3.8-cm diameter and 7.6-cm height) with MP compaction energy. The specimens were cured in a moist chamber at a temperature of 20 ± 3 °C and a relative humidity of 95% ± 5% until the testing ages: 0, 3, 7, 28, and 90 days. Three specimens were tested for every soil sample at every age. The uniform breaking speed in the press was 1 mm/min.

#### California Bearing Ratio (CBR) test

The California Bearing Ratio (CBR) index is an indirect measure of the soil shear strength and depends on the moisture content and level of compaction. The CBR test is therefore used to evaluate the bearing capacity of compacted soils such as embankments, pavement layers, and subgrade, and its results define the bearing capacity of soil samples, as well as swelling after a period of immersion in water, according to ASTM D1883-21 (2021). After mixing the sample and determining its OMC and MDD with the Proctor Compaction Test, it was dynamically compacted in a standard mold in three layers of similar thickness by applying 15 blows per layer with a 2.5 kg rammer. Subsequently, the specimens were immersed in water for 96 h with a surface overload of 4.5 kg, taking strain readings with dial gauges every 24 h. The CBR test was then performed with an automatic press, whose maximum load cell force measurement and penetration piston speed were 50 kN and 1.2 mm/min, respectively.

#### Volumetric stability tests

Spanish regulations (Ministerio de Fomento 2015) approve the use of stabilized soils in the foundation and the core of embankment fillings, if their free-swelling is not in excess of 1.5%. Therefore, with the aim of evaluating the volumetric stability of the 9 soil samples, free-swelling tests were conducted in an oedometer according to ASTM D4546-21 (2021). This test consists of measuring the deformation experienced by a laterally confined soil specimen subjected to a vertical pressure of 10 KPa and inundated with water, until the strain reaches the equilibrium. The free swelling is expressed as the percentage increase in height of the specimen with respect to its initial height. Specimens with a diameter of 4.95 cm and a height of 2 cm, compacted with MP conditions were used.

#### Direct shear test

This test consists of determining the strength parameters, cohesion (*c*′), and internal friction angle (*Φ*'), of a soil sample subjected to shear stress, according to EN ISO 17892-10 (ISO 2018). The test was performed on three cylindrical specimens, 20 mm in height and 50 mm in diameter, of each soil sample. These specimens were compacted with the MP conditions and then subjected to a primary consolidation process for 24 h with a predetermined vertical pressure to facilitate drainage. Later, the specimens were placed in the direct shear box, in which vertical pressure was maintained, and a horizontal shear force was applied in such a way that the specimens were broken along a predefined horizontal plane. The shear speed was determined according to the consolidation-phase results. The test was repeated for three different vertical pressures: 100 KPa, 200 KPa, and 300 KPa. In each determination, both the vertical and the horizontal strains and the vertical and the shear load cell data were measured, so the applied tangential and normal stresses were determined. A consolidated-drained test was performed in this research, in which drainage took place in both the primary consolidation phase and the failure phase. For this purpose, the rupture velocity was sufficiently slow, so that no interstitial pressures were generated.

#### pH evolution

pH measurements were performed over the curing time of the soil samples according to EN ISO 10390:2021 (ISO 2021) to determine their pH evolution. Two test specimens of each mix were prepared with the OMC and MP compaction energy to perform this test. Subsequently, the specimens were preserved in a moist chamber during different curing ages (0, 3, 7, 14, 21, 28, 56, and 90 days). After these curing periods, 5 g of soil, which were obtained from the produced specimens, and 25 ml of distilled water were mixed and subjected to agitation for 1 h, and subsequent repose during another hour. After these times, the pH measurements were taken. The pH-determination samples were prepared with a mortar and pestle to obtain the maximum specified size, avoiding the use of an oven, due to the fact that water content or temperature variations can affect the products formed during the hydration reactions and, therefore, their physical behavior (Boardman et al. 2001).

## Results and discussions

### Atterberg limits

**Fig. 4:**
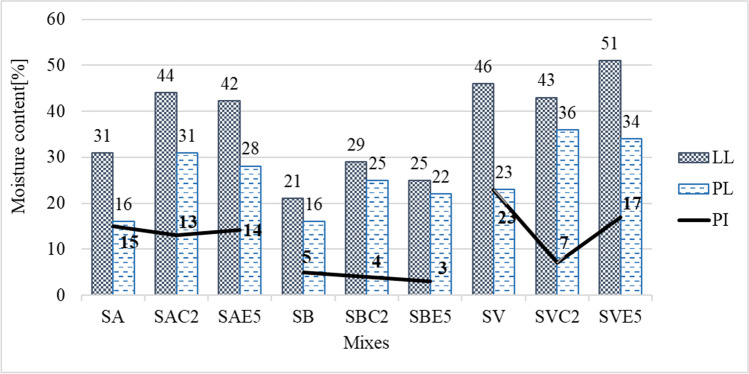
Atterberg limits of natural soils and their mixes with lime and LFS

The results obtained for the liquid limit (LL), the plastic limit (PL) and the plasticity index (PI) are depicted in Fig. 4. The control mixes with 2% lime showed an increase in LL of 13 percentage points for SAC2 and 8 points for SBC2, while SVC2 exhibited a reduced LL by 3 points. In all cases, the PL increased with the addition of lime, obtaining values of 31%, 25%, and 36%, for the SAC2, SBC2, and SVC2 mixes, respectively. As a result, the plasticity index of the mixes was slightly reduced, trend also found in other studies (Wild et al. 1998; Boardman et al. 2001; Rahmat and Kinuthia 2011; Manso et al. 2013). The plasticity reduction was more prominent in the SV soil, in which the PI was reduced in 16 percentage points as the PI decreased from 23 to 7% when stabilizing with lime (SVC2 mix).

The reduction of the PI when lime was added was due to cation exchange that occurs when the cations in the double diffuse layer of the clay particles can be replaced with calcium ions (Ca^2+^) present in the lime cation exchange, which led to a decrease in the LL and an increase the PL of the clay. However, depending on the mineral structure of the clay, an increase in the liquid limit (LL) could indeed be possible, as observed in kaolinite and quartz soils (Bell 1996; Boardman et al. 2001; Rahmat and Kinuthia 2011). Dash and Hussain (2012) proposed that the LL behavior in lime-treated soils can be categorized into three phases: initially, a decrease due to rapid reduction in the thickness of the diffuse double layer; secondly, flocculation result in a LL increase, and finally, pozzolanic reactions generate gelatinous material that retains water, leading to an improvement in LL. Those short-term reactions meant that any changes can be immediately appreciated after mixing the binder with the soil. As a result of these modifications, an improvement in the workability of the soil is obtained (Sherwood 1993).

The tendencies of both the LL and the PL were similar both for the LFS mixes and for the soils stabilized with lime, which increased with respect to the LL and the PL of the natural soils, which in turn led to a slight decrease in the PI. However, the LL reduction of the SVC2 mix differed in trend from the other mixes, which could be due to a low percentage of lime addition for this soil and without curing time, so the changes could be attributed to cation exchange alone. Dash and Hussain (2012) obtained similar results with a residual soil having similar plastic characteristics than SV. Less plastic soils, such as the SB soil, with compounds such as kaolinite or illite, showed neither rapid nor important changes. The more plastic the soil, and higher its specific surface and the faster the changes it underwent (Bell 1996; Boardman et al. 2001), as with the SA and SV soil mixes.

### Modified compaction test

Table 5 shows the results of the Optimum Moisture Content (OMC) and Maximum Dry Density (MDD) obtained in the Modified Proctor (MP) test.

**Table 5:** MP test of natural soils and lime- and LFS-stabilized soils

Mixes	SA	SAC2	SAE5	SB	SBC2	SBE5	SV	SVC2	SVE5
MDD (Mg/m^3^)	1.86	1.84	1.84	2.05	2.03	2.03	1.75	1.73	1.73
OMC(%)	10.30	10.80	10.80	8.40	8.90	8.90	13.50	14.50	14.50

**Table 6:** Direct shear results of the 9 soil samples

Curing age	Soil A	*c*′ (KPa)	*Φ*′ (°)	Soil B	*c*′ (KPa)	*Φ*′ (°)	Soil V	*c*′ (KPa)	*Φ*′ (°)
0 days	SA	19	34	SB	42	29	SV	45	29
7 days	SAC2	59	30	SBC2	83	24	SVC2	49	27
28 days	SAC2	59	32	SBC2	84	27	SVC2	63	22
7 days	SAE5	54	28	SBE5	49	36	SVE5	51	41
28 days	SAE5	79	34	SBE5	76	32	SVE5	76	32

It was found that the binders slightly modified the reference values obtained for the natural soils, because low substitution percentages were used and the fact that the determination was conducted immediately after mixing, which represented little or no variation in relation to the natural soil. The results for the mixes with lime and LFS were identical to each other and showed, in general, a decrease in the MDD and an increase in the OMC compared to the values for natural soils, which in turn translated into a light flattening of the Proctor curve. These results were in accordance with the existing literature (Bell 1996; Rahmat and Kinuthia 2011; Ghobadi et al. 2014; Diniz et al. 2017). The increase in the OMC was 0.5 percentage points higher compared to the untreated soils in the SAC2, SAE5, SBC2, and SBE5 mixes (10.80% and 8.90%, compared to 10.30% and 8.40%, for the SA and SB soils, respectively). There was a 1% increment of the OMC (14.50%) with respect to the SV natural soil (13.50%) in the SVC2 mix. The MDD variations were not very significant, with reductions of 0.02 Mg/m^3^ in all mixes.

The slight changes arising from the addition of lime may be attributed to the flocculation that occurs when the diffuse double layer in the cation exchange decreases, which is eased by the existing high electrolyte concentration and the high pH induced by lime addition. Flocculation and agglomeration generate an increase in void volume and thus a reduction in the MDD (Rahmat and Kinuthia 2011). This decrease in MDD can also be attributed to the formation of cementitious products, which reduce the compactability of a treated soil (Abdelkader and Hamdani 1985).

Different investigations on LFS-stabilized mixes have a priori exposed contradictory results. Montenegro et al. (2013), who carried out studies with two types of soils, found that the trend of one of the soils mixed with LFS was similar to the trend recorded for lime, while the other soil, with a higher percentage of fines and higher PI, presented a slight increase in the MDD and an OMC decrease when the percentage of LFS was increased. Nevertheless, Brand et al. (2020) proved that an increase in the percentage of LFS generates increments in OMC and MDD. Manso et al. (2013) obtained results similar to the ones of this study, with minor variations in the OMC and the MDD when adding both lime and slag. Finally, Lopes et al. (2022), who worked with a clayey soil and a sandy soil, found that in both cases the increase in the percentage of LFS in the mixes led to a decreasing tendency in the OMC and a small increment in the MDD, which was attributed to the filler size, caused by the lower size of the LFS particles and their morphology. It therefore appears quite clear that the variations in the OMC and the MDD when stabilizing with LFS depend on the nature of the soil and the LFS.

### Unconfined compressive strength

**Fig. 5:**
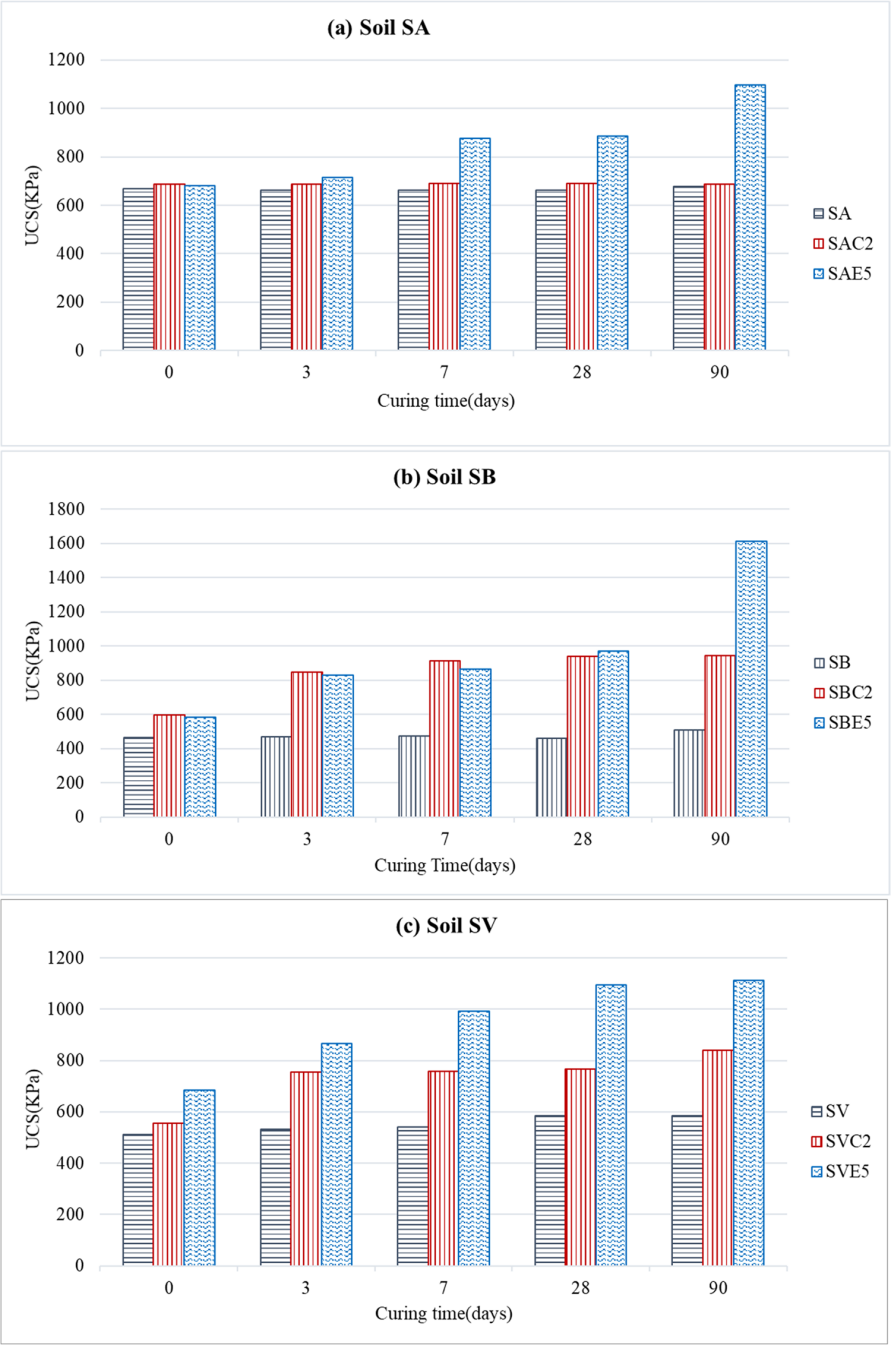
UCS vs. curing time: **a** SA mixes; **b** SB mixes; **c** SV mixes

Figure 5 shows the evolution of the unconfined compressive strength (UCS) over time of all the mixes tested at 0, 3, 7, 28, and 90 days. The UCS of a stabilized soil mainly depends on four different factors, which must also be considered when stabilizing with LFS:

- First, the mineralogical composition of clay is a fundamental factor in the strength development of stabilized soils (Eades and Grim 1960). The mineralogical structure conditions the level of activity on the surface of the clay particle, which is called cation exchange capacity. Clay soils such as kaolinite have a relatively low cation exchange capacity compared to other expansive clay soils, such as montmorillonite, which have a high cation exchange capacity (Bell 1996). This property affects the short-term behavior of the mixes, but Bell (1996) suggested that it is not an important factor in terms of long-term strength development. With respect to chemical composition, clay soils with higher percentages of Si_2_O_3_ react more strongly with lime or other stabilizers. For example, reactions with montmorillonite clay are faster than with kaolinitic clays, while illite and chlorite are much less reactive than montmorillonite (Bell 1988).
- Second, the percentage of stabilizer added to the clayey soil is also a determining factor. In the various studies conducted on lime-stabilized soils (Eades and Grim 1960; Bell 1988; Rogers and Glendinning 2000), it has been found that when lime is added to the soil in the presence of water, the first thing is to satisfy the soil’s affinity for lime. The calcium ions in lime are adsorbed by the clay minerals, so they are not available for pozzolanic reactions until that affinity is satisfied (Bell 1988). The percentage of lime at which this process is completed is called the fixation point, and its effects improves the workability of the soil. However, only the excess lime over the fixation point is used in the cementation processes that confer increased strength (Bell 1996). In previous research (Eades and Grim 1966; Boardman et al. 2001), it has been suggested that an insufficient amount of lime may mean that cation exchange remains incomplete and that the physical effects of flocculation are not forthcoming in the short-term, or that there is insufficient lime for pozzolanic reactions to occur in the long-term (Rahmat and Kinuthia 2011).
- Third, the curing process has also to be considered. It is clear that the effect of the stabilizing binder on strength development is a function of time, temperature, and relative humidity (Mitchell and Hooper 1961). Time-dependent pozzolanic reactions lead to progressive strength development throughout the curing time (Bell 1996). In the short term, strength improvement is attributed to the formation of poorly ordered cementitious products surrounding the clay particles. The strength gain in the long term is however associated with the gradual crystallization of new structurally ordered minerals (Bell and Coulthard 1990). This crystallization is caused by cementing processes, due to the slow hydration of di-calcium silicate and pozzolanic reactions between the clay fraction of soils and lime. Therefore, all samples have to be kept under the same curing conditions to exclude their effect on the comparative strength analyses.

Finally, the moisture content of the soil is another factor to be controlled. According to Bell (1988), soil-lime mixes compacted at moistures above the OMC, for short curing periods, develop higher strengths than those compacted below the OMC. On the other hand, Bell (1988) also indicated that, if the amount of mixing water is insufficient, the hydration processes necessary for the cementitious reactions do not occur, and in that case, the maximum strength increases are developed after a few days. Therefore, the compaction of the samples was based on the OMC, so that the moisture content had no effect on the strength results.

Figure 5a shows the null effect on UCS of lime in the SA soil, regardless of the curing time. The observed changes in LL and PL indicated that the cation exchange was initiated, which produced the changes in the plastic properties of the mixes in the first curing ages. Small amounts of lime, generally between 1 and 3% depending on the amount and type of clay minerals present in the soil, are sufficient to cause these plasticity changes (Bell 1996). However, low percentages in certain soils never achieved significant strength improvements, as can be noted in the work of Eades and Grim (1966). This behavior may in the case of the SA soil be explained in terms of its chemical composition (Table 2). SA had a low percentage of Si_2_O_3_ (20.52%) and a not very high percentage of Al_2_O_3_ (8.27%), apart from the fact that the alkalinity conditions were not the most optimal (pH never exceeded 11.72 points, as shown in the “California Bearing Ratio (CBR)” section). Both aspects point to a very low pozzolanic reaction and no formation of calcium silicate hydrate (CSH) and calcium aluminate hydrate (CAH) cementitious gels, which might otherwise have strengthened the mixes. In contrast, the addition of LFS to the SA soil resulted in a continuous improvement of the UCS from 7 days (875 KPa), so UCS values of about 1096 KPa were reached at 90 days, which were 1.6 times higher than those of the untreated soil. In this case, the development of high strengths required long curing times for the cementitious reactions to occur, mainly related to the periclase contained in LFS (Table 3). It is remarkable that the strength of the soil-LFS mix increased by 32% after 7 days of curing, a trend that increased up until 90 days of curing when improvements of almost 62% were reached.

The addition of stabilizers to the SB soil resulted in an improvement of UCS since the first day, both with LFS and lime (Fig. 5b), with a slight upward trend up until 28 days of curing. The UCS of the SBE5 mix (1612 KPa) presented a 188% higher strength at 90 days than the UCS of the untreated soil (559 KPa), which was also higher than the improvement of 92% for the SBC2 mix (945 KPa). This soil with minerals such as kaolinite and illite exhibited remarkable and quick increases in UCS, which are similar to the results of other studies (Bell 1996; Boardman et al. 2001), indicating that the cementitious reactions were properly produced. In particular, the SBE5 mix obtained very successful results due to the reaction of lime, alumina, and silica generated slight hydrated cementitious products, such as CSH and CAH, which were responsible for the increased long-term strength of the mix.

The SV soil presented a high percentage of SiO_2_ (45.25%), Al_2_O_3_ (10.85%), and MgO (8.25%), all compounds that in an alkaline environment and in the presence of water lead to improvements of the mix when adding a stabilizer. Thus, the strength behavior of the SV soil mixed with lime improved considerably with respect to the natural soil (Fig. 5c). This improvement was mostly notable on the third day of curing, when it experienced an improvement of 33%, as the improvement trend subsequently softened, reaching a maximum of 840 KPa at 90 days, which represents an increase of 44% with respect to the untreated soil (585 kPa). When the amount of lime is small, strength improvements are not significant, even for long curing periods. In these situations, lime is mostly used to satisfy the initial requirement of the soil, the cementation is quite weak, generating little strength gain (Eades and Grim 1966; Dash and Hussain 2012). With respect to the LFS-stabilized mix, the improvement was much higher, registering an increase of 34% on the same day of mixing, increasing progressively throughout the curing time, to reach a value of 1114 KPa at 90 days, which represents an improvement of over 90%. These results demonstrate the cementitious capacity of LFS, which involves aggregating particles and providing higher strength (Papayianni and Anastasiou 2012; Diniz et al. 2017). The strength improvements observed over the curing time are associated to the slow hydration processes and pozzolanic reactions of the clay with the lime present in LFS (Manso et al. 2013; Lopes et al. 2022).

It has been found that the improvement in strength is highly dependent on the type of soil to be stabilized, the importance of the curing time to allow the development of pozzolanic reactions to acquire maximum strength, and the need to determine the optimum binder content for a better stabilization response. Based on the results of this study, the use of LFS shows a better strength performance than the use of lime, with the added advantage of the environmental benefit of reusing a waste material.

### California Bearing Ratio (CBR)

**Fig. 6:**
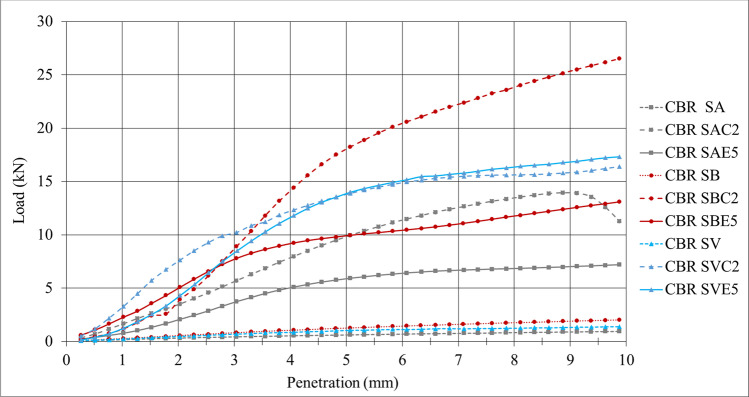
Penetration vs. load results in CBR tests of the 9 soil samples

**Fig. 7:**
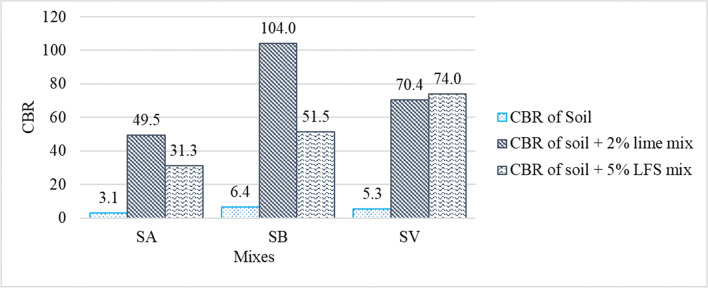
CBR results of the 9 soil samples

Figures 6 and 7 show the CBR results obtained for the 9 soil samples after 4 days of immersion in water. Their deformations were measured every 24 h during this period of time.

All the soils under study showed significant increases in CBR when mixed with both 2% lime and 5% LFS. The improvements in the SB and SV soils were more remarkable than in the SA soil. These values are concordant with the values reported in the existing literature, which indicated that the CBR index increased immediately after adding lime/LFS to the soil and continued to increase with the curing time (Bell 1996).

In the SA and SB soils mixed with lime, the CBR increases (49.5% for SA and 105% for SB) were higher than when LFS was used as a stabilizing agent (31.3% for SA and 51.5% for SB). The results for the SV soil were very similar for both binders (70.4% with lime and 74% with LFS). Similar results were reported in other comparative works on stabilization with lime and LFS (Manso et al. 2013) and in soil stabilization with basic oxygen furnace slag (BOFS) (Diniz et al. 2017), in which the CBR results for stabilizations with lime were in general slightly higher than for stabilizations with slag. This performance is fundamentally attributed to the fact that the reactions in the mixes with slag require lengthier curing times than 4 days (Boardman et al. 2001; Manso et al. 2013; Diniz et al. 2017; Lopes et al. 2022).

It can be stated that the stabilization with 5% LFS resulted in adequate increases in CBR which exceeded the minimum soil bearing capacity values required by the regulations for soil stabilization and the formation of road embankments (Thompson, 1969; Ghobadi et al., 2014). While the results in some cases are slightly inferior to those obtained in stabilization with conventional binders, they remain comparable and offer the distinct technical advantage of providing a more sustainable solution for soil stabilization. Previous studies have reported that the main effects of LFS on the CBR index of soils were attributed to the hydration of aluminates and the reaction of free lime and periclase with the clay fraction, improving soil compaction and flocculation (Manso et al. 2013). Additionally, during the hydration and carbonation processes of the lime and magnesia present in the LFS, products such as portlandite, brucite, calcite, and magnesite appear, which are larger in volume than the former, filling the pores and generating a denser soil matrix, thereby improving the compaction and bearing capacity of the mixes (Ortega-López et al. 2014; Lopes et al. 2022).

### Volumetric stability tests

**Fig. 8:**
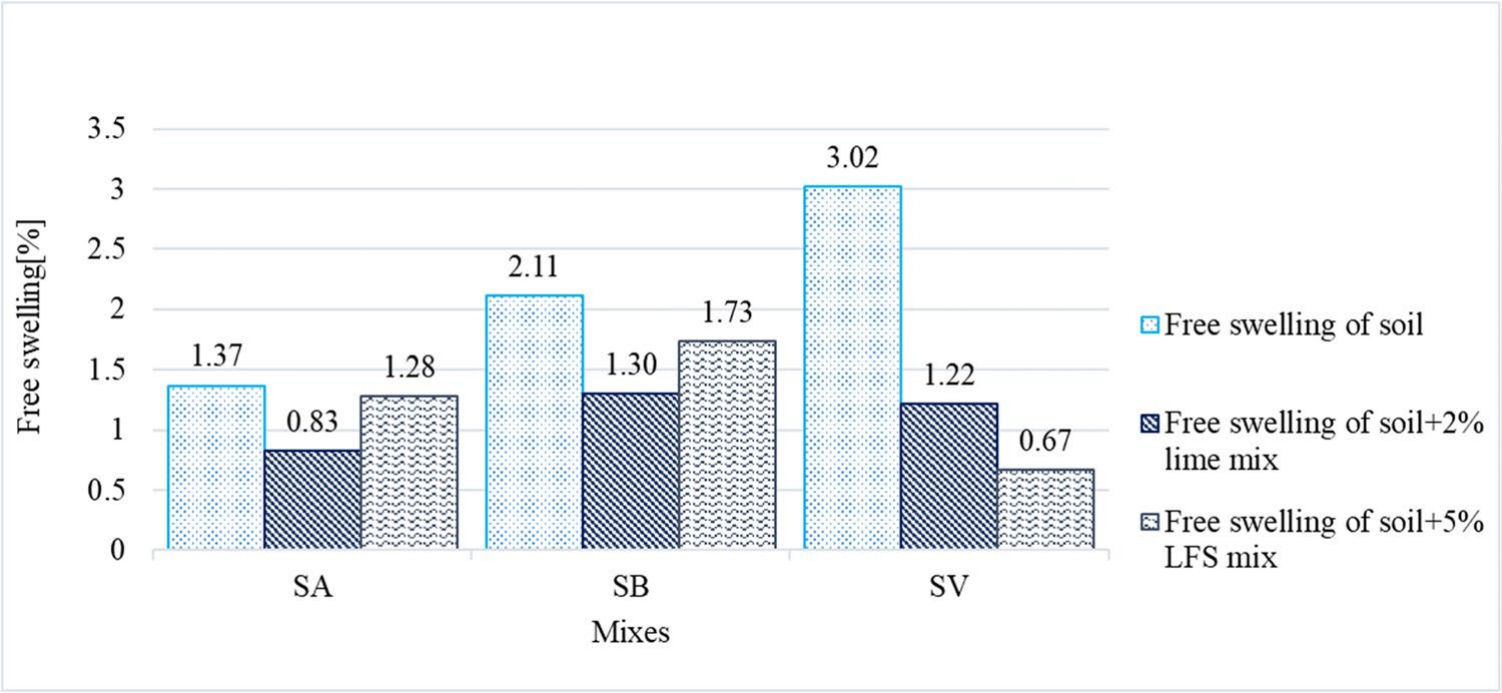
Free-swelling results of the 9 soil samples

**Fig. 9:**
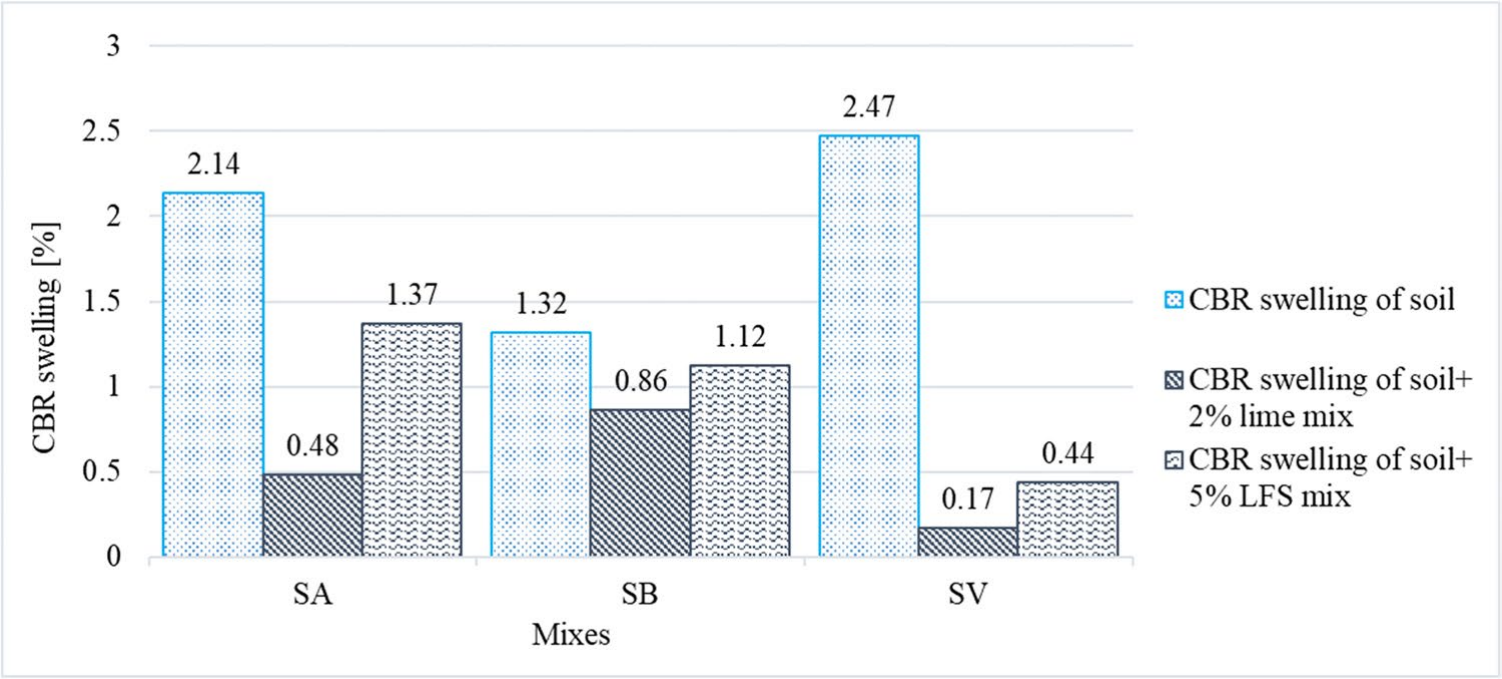
CBR-swelling results of the 9 soil samples

The volumetric properties of the soils and their stabilized mixes were evaluated in oedometer free-swelling tests (Fig. 8) and in CBR-swelling tests, during which the soil samples were immersed in water at room temperature for 4 days (Fig. 9).

The free swelling (Fig. 8) of natural soils SA (1.37%) and SB (2.11%) measured in an oedometer was lower than the maximum value recommended (3%) in the Spanish standards for soils directly stabilized with lime or cement (Ministerio de Fomento 2015). In case of exceeding this maximum, the regulations establish that the free-swelling test should be performed on the stabilized soil after 24 h in a moist chamber. This procedure was followed with the SV soil, with an initial free swelling of 3.02%.

The SA and SB soils showed a greater swelling reduction when mixed with lime (0.83% for the SA soil and 1.30% for the SB soil) than when using LFS (1.28% for the SA soil and 1.73% for the SB soil). As the free-swelling measurements were conducted immediately after mixing, without curing time, most chemical reactions were not present, especially those related with the MgO of LFS, which usually require more curing time. Therefore, the swelling decrease in the LFS-stabilized mixes (SAE5 and SBE5 mixes) was not high (Ortega-López et al. 2014; Santamaria et al. 2018; Montenegro-Cooper et al. 2019).

However, the SV mixes, that were in moisture chamber 24 h prior to the test, as per the reference standard (Ministerio de Fomento 2015), resulted in higher swelling decreases both in its mix with lime (1.22%) and with LFS (0.67%). It seems that this short curing period clearly favored the stabilization reactions (Seco et al. 2021; Lopes et al. 2022).

The CBR swelling (Fig. 9) of natural soils (2.14% for SA, 1.31% for SB, and 2.47% for SV) was reduced with both types of binders, the most outstanding decrease being that of the SV soil (0.17% and 0.44% for SV mixes with lime and LFS, respectively). Furthermore, the reduction in the CBR swelling was slightly more pronounced for the three soils within the lime-based mixes rather than the LFS mixes, which can be justified due to the longer curing times needed for LFS-based stabilization reactions (Boardman et al. 2001; Manso et al. 2013; Diniz et al. 2017; Lopes et al. 2022).

The results of this study are similar to previous results obtained by other authors. They concluded that, in spite of the expansive nature of LFS (Manso et al. 2013; Montenegro et al. 2013; Ortega-López et al. 2014; Montenegro-Cooper et al. 2019; Lopes et al. 2022), the use of LFS in small quantities in flexible matrices, such as a soil, caused no expansion problems, although the lime and magnesia of the LFS helped to flocculate the clay particles, which led to decreased plasticity and swelling in the first curing ages. For a more complete evaluation, free swelling may be evaluated at different curing ages, as in the study of Lopes et al. (2022), or through an accelerated-aging test, as reported in Ortega-López et al. (2014).

### Direct shear test

**Fig. 10:**
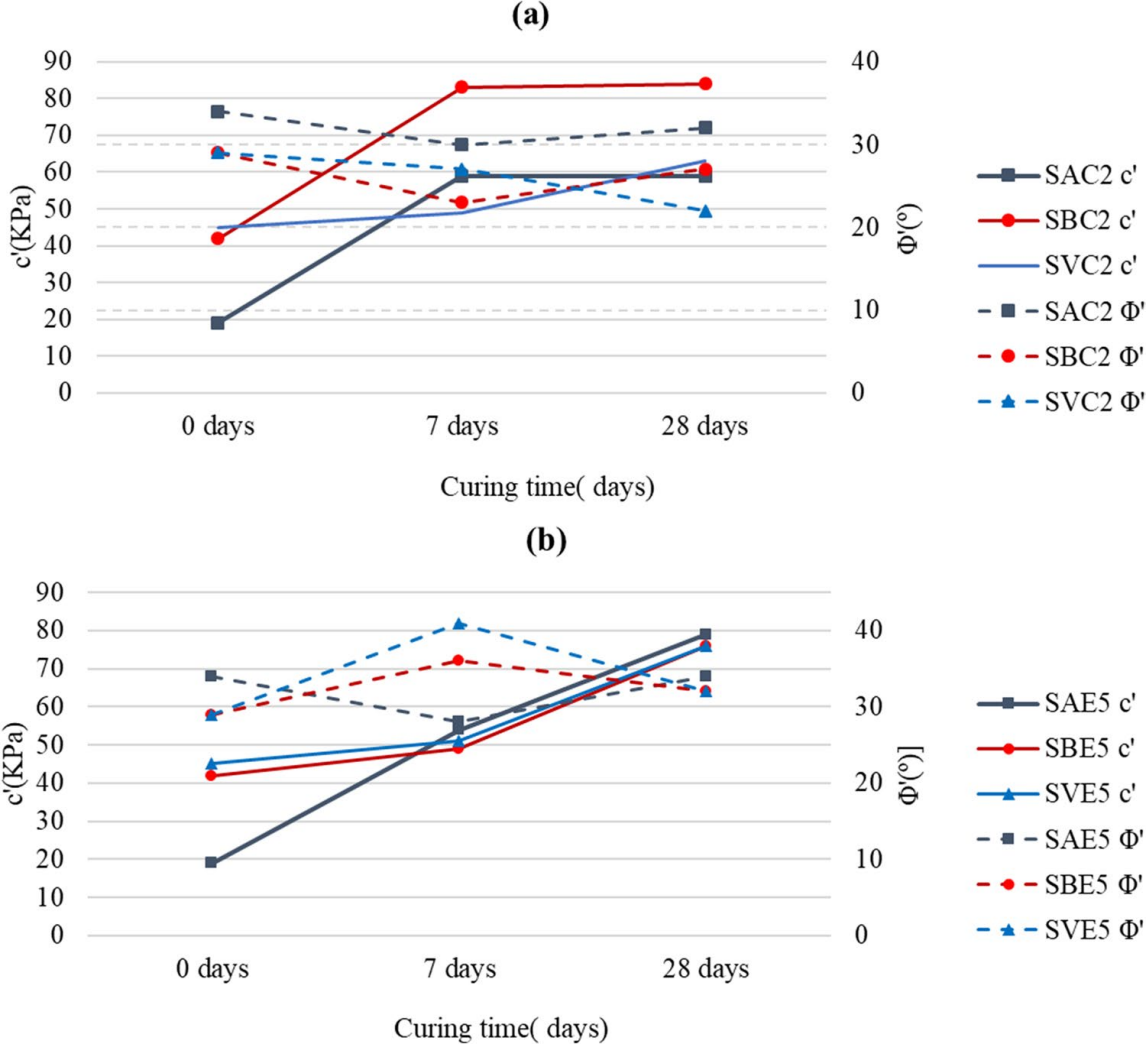
Evolution of *c*′ and *Φ*′ over curing time: **a** lime-stabilized soils; **b** LFS-stabilized soils

Table 6 and Fig. 10 show the results obtained from the direct shear test of natural soils and their respective mixes with lime and LFS: cohesion (*c*′) and effective angle of shearing resistance (*Φ*′). Initially, high values of cohesion and effective angle are indicative of good shear strength or resistance to shearing stresses, especially for soil cohesiveness. Nevertheless, when the soil is a clay, with high cohesion, such as the soils presented in this study, there are other parameters, shown in previous sections, rather than only the direct shear results, which must be considered for determining its final behavior. As M.R. Thompson concluded (Thompson 1969), the shear strength parameters used in the design of lime-soil mix pavement layers were not probably critical.

In the case of the SA soil (*c*′ = 19 kPa; *Φ*′ = 34°), the addition of 2% lime (SAC2) implied an increase in effective cohesion (59 kPa for both 7 and 28 days) and a slight decrease in the friction angle (30° and 32°, for 7 and 28 days, respectively). However, the addition of LFS led to a considerable increase in cohesion over time (54 kPa and 79 kPa for 7 and 28 days, respectively), mainly related with the cementation processes. With respect to the friction angle, although in the first days it decreased (28° at 7 days), it recovered its initial values at 28 days (34°).

The behavior of the SB soil was similar. The natural soil had a high cohesion value (42 kPa) and a medium friction angle (29°). When lime was added to the soil, the cohesion increased by 69% (83 kPa), and the friction angle decreased (24°) after seven days, although the cohesion (84 kPa) and the friction angle (27°) values after 28 days were almost identical to those obtained after 7 days. With respect to the LFS-stabilized mix, the changes followed the same trend. After a curing period of 7 days, the cohesion and the friction angle had increased by 17% (49 kPa) and 24% (36°), respectively. When the curing period was extended to 28 days, the cohesion reached a value similar to lime-stabilized mix (76 kPa) and the friction angle underwent a slight decrease (32°).

The strength parameters of the SV natural soil showed a high cohesion value (45 kPa) and a medium internal friction angle (29°). The cohesion (49 KPa) improved slightly at 7 days of curing and even more at 28 days (63 KPa) when adding lime. On the contrary, the friction angle suffered a little reduction at 7 days (27°), but after 28 days of curing, its value dropped by 25% with respect to the value of the natural soil (22°). The behavior of this SV soil when mixed with LFS was analogous to that of lime. Cohesion slightly increased in the first 7 days (51 kPa), and at 28 days, it had increased 69% with respect to the untreated soil (76 kPa). Friction initially increased by 41% (41°), and after 28 days, it tended to decrease (32°), so only a slightly higher value (10% better) than that of the natural soil was reached.

There are few previous publications on the evolution of shear strength parameters in soils stabilized with either lime or some type of slag, and the results are often contradictory. Thompson (1969) analyzed the evolution of the shear strength properties of four soils stabilized with 3 and 5% lime, and concluded that mixes substantially increased the shear parameters, determining large improvements in cohesion and small increases in friction angle, and that these improvements were greater as the curing period increased. In the study of Ghobadi et al. (2014) on soil stabilization with 7% lime, the effects of pH on the shear strength parameters at 30 days were analyzed. The author found that the cohesion of the mix decreased and the friction angle increased with respect to the untreated soil. However, Boardman et al. (2001) measured the evolution of undrained shear strength with a shear vane apparatus, determining that the cohesion value increased in stabilized soils with the curing time, regardless of the percentage of lime in use. The results obtained in the lime-soils mixes in the present investigation (Fig. 10a) are in line with those in the aforementioned study, as cohesion increased with respect to the value of the untreated soil and the friction angle slightly decreased after a curing period of 28 days.

Regardless of the results of the LFS-stabilized mixes (Fig. 10b), the final trend after 28 days of curing was a substantial increase in cohesion, while the friction angle also moderately improved. However, the temporal evolution of those parameters was variable depending on the natural soil, which decreased or increased in the intermediate stages of curing (7 days), results also observed in lime-stabilized mixes. These results are in accordance with the studies of Akinmusuru (1991), who showed the evolution of the shear strength parameters with respect to the percentage of blast furnace slag (BFS) added to the mix. In that study, the higher percentage of fines, due to the increased addition of BFS, led to an important improvement in cohesion, but to a smooth decrease in the friction angle. Mahesh Bhat and Nayak (2021) also studied the 28-day strength parameters in soils stabilized with lime, GBFS, and with a mix of GBFS and lime. In all cases, there was a large increase in the cohesion values and a notable increase in the friction angle.

### pH evolution

The evolution of pH over time can be interpreted as an index of the rate of pozzolanic fixation of lime and magnesia in clays (Manso et al. 2013). In fact, Bell (1988) indicated that the strength increase of stabilized soils is proportional to the reduction in their pH value over the curing time. The combination of water and lime in adequate quantities generates a highly alkaline environment that favors the decomposition of the clay particles and the liberation of silica and alumina. A pozzolanic reaction cannot occur without the release of silica and alumina from the clay. Consequently, it is evident that the study of pH and its evolution over time provides information on the chemical reactions within the soil.

**Fig. 11:**
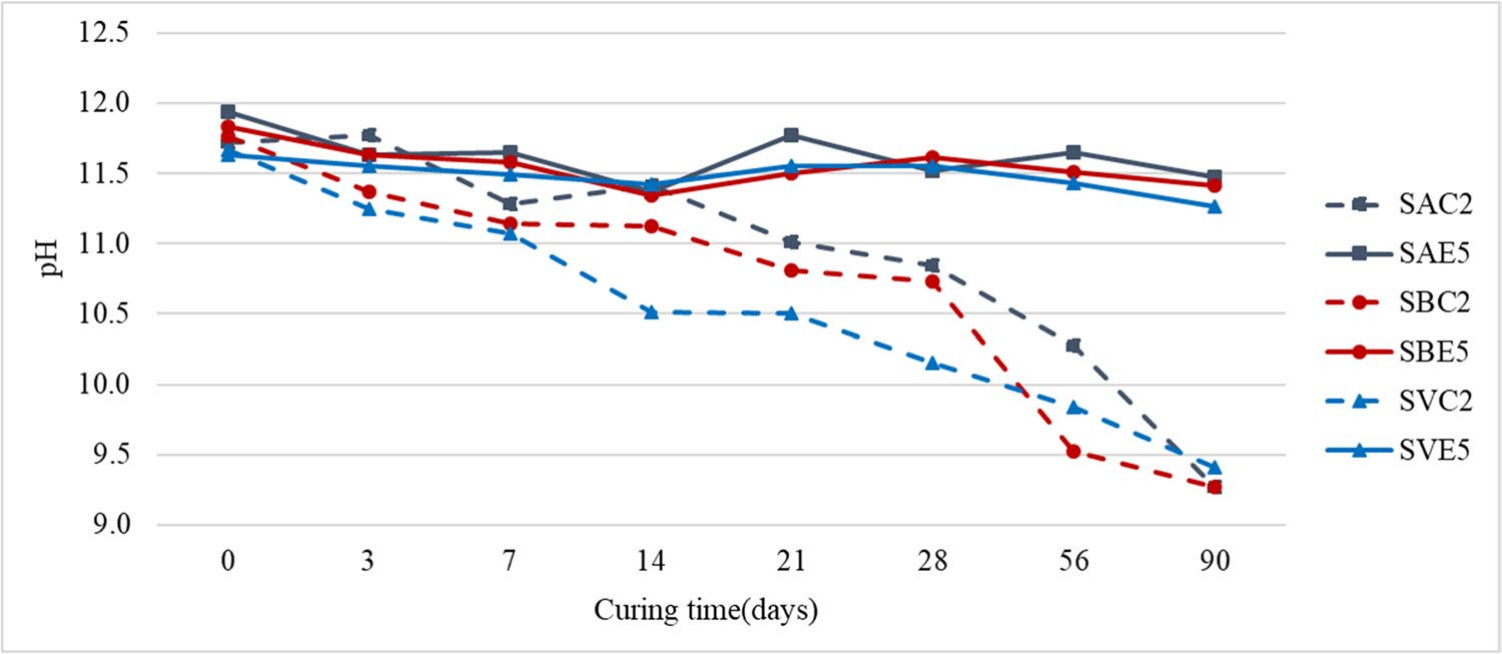
Evolution of pH over the curing time

**Fig. 12:**
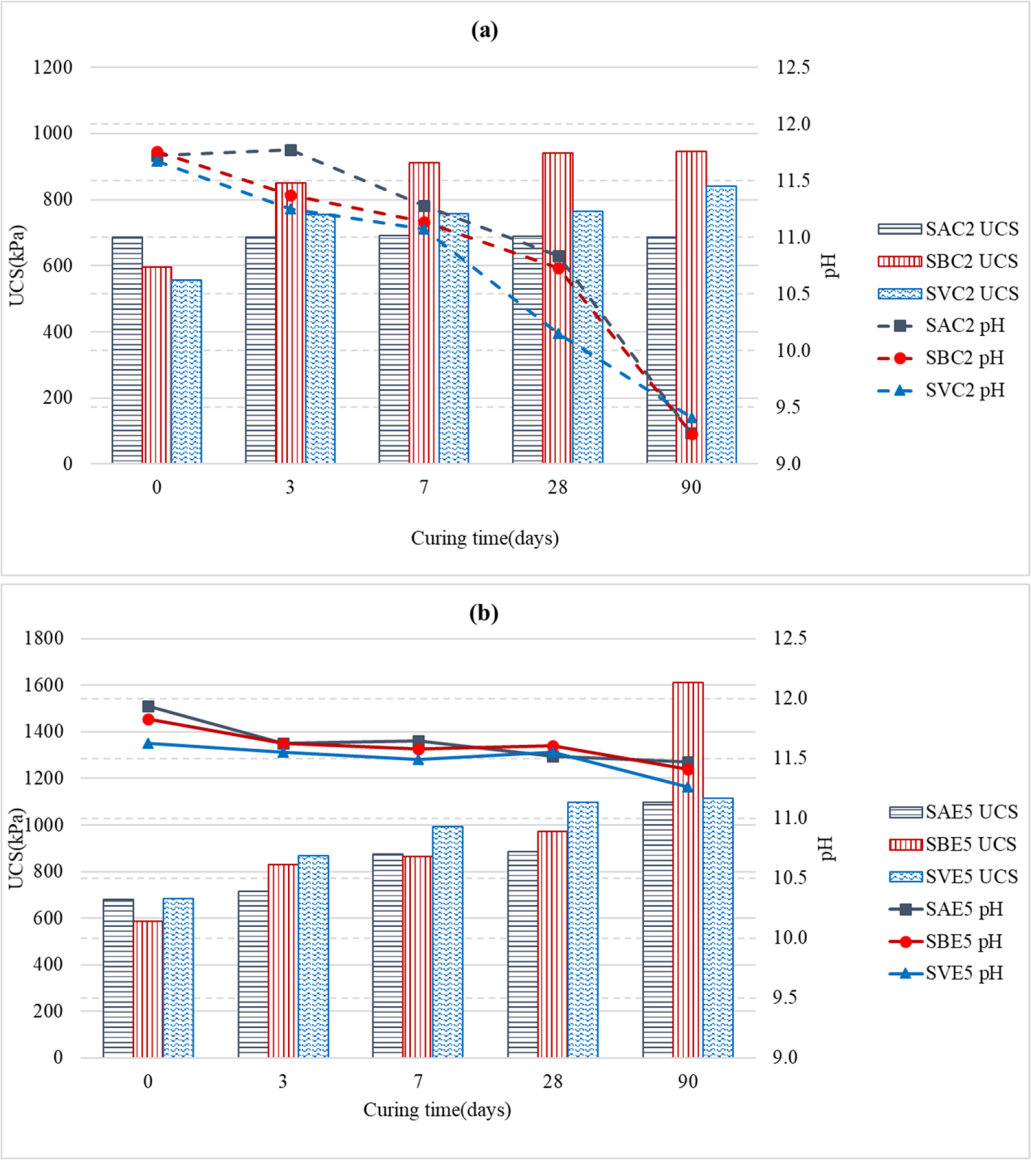
Evolution of pH and UCS over the curing time: **a** lime-stabilized soils; **b** LFS-stabilized soils

The addition of lime or LFS to a soil usually increases the pH to values above 11.5. In this high alkaline medium, clays are unstable and can solubilize their components. The pH value of the interstitial fluid in the voids should remain at around 12.4 to achieve the best reactivity results of soils with stabilizers, because, at these values, the solubility of silica and alumina ions is very high (Bell and Coulthard 1990). In this way, silica and alumina are released and react with calcium from the lime to form CSH and CAH, which will continue to form as long as high alkalinity conditions persist in the soil (Diniz et al. 2017; Lopes et al. 2022). For these reasons, a study was performed on the evolution of pH in the 9 soil samples to correlate the pH and UCS values (Fig. 12). Table 7 contains the pH of the natural soils and the two binders (lime and LFS), and Fig. 11 shows the pH of the 6 soil-stabilized mixes during the curing period.

**Table 7:** pH of natural soils and binders

	Soil SA	Soil SB	Soil SV	Lime	LFS
pH	8.65	8.58	8.88	12.67	12.62

It can be noted from Table 7 that the three soils were alkaline and both LFS and lime had an extremely basic character. In all cases, the soil stabilization with both binders therefore resulted in an increased pH of 11.6 (Fig. 11). None of the pH readings reached 12.4 points, a value established by Eades and Grim (1966) as the target for determining the optimum percentage of lime in soil stabilization. This result indicates that the amounts of lime or LFS added in this study were, in general, less than the optimal amount. Goodarzi and Salimi (2015) tested the pH increase in soils stabilized with different percentages of granulated blast furnace slag (GBFS) and basic oxygen furnace slag (BOFS) and found pH values above 12.5 in mixes with 30% BOFS. However, this evolution varied with the type of slag, the hydraulic index of which was determined by its chemical composition.

The tendency of pH evolution over time was quite clear (Fig. 11), but pH and UCS results were different for lime and LFS:

- On the one hand, as can be observed in Fig. 12a, when soils were initially mixed with lime, the mixes increased their pH by around 3 points (pH values of 11.72 for SAC2, 11.68 for SBC2, and 11.83 for SVC2), which indicated that there was a basic environment necessary to activate the chemical processes. From that point, the pH continued decreasing until values of around 9.25 at 90 days. The SBC2 and SVC2 mixes showed similar trends, as in the first 7 days, they showed a pronounced decrease, with a slope of around 17%, which subsequently moderated, with slopes close to 10%. In parallel, these mixes showed the greatest improvement in strength in the first 7 days, confirming that the highest strength increases, as Bell (1996) affirmed, occurred in this period when the cementitious reactions were most active. Nevertheless, the behavior of the SAC2 mix is different. Between the beginning of the process and up to 14 days, the pH underwent a staggered decrease, with periods of almost no variation, between 0 and 3 days and between 7 and 14 days, and periods with pronounced peaks. From day 21 of curing, the pH decreased with similar slopes in all the other mixes (10%). Based on these pH results and the above-mentioned plasticity variations, it could be stated that exchange cations and possibly some flocculation were produced. However, the low increase in strength (3%) in the SAC2 mix with respect to the untreated soil, SA, throughout the curing time, might make it reasonable to think that the cementing process was not initiated. This could be justified by the low percentage of SiO_2_ and Al_2_O_3_ in the system (Bell 1988). These results are in contrast with the study of Boardman et al. (2001), who noted pH values that were almost maintained (small variations for 300 days) in two different types of soils mixed with different percentages of lime. In the study of Eades and Grim (1966) where soils were stabilized with different percentages of lime, it was noted that the increase in UCS was high with high percentages of lime (5%, 7%, and 9%), while the pH values remained above 11.35. For low percentages of lime (1% and 3%), the final increase of strength was also low, but with a constant upward tendency, maintaining a pH value above 11.05, a result that corroborates the need to maintain a high pH, so that the cementitious processes can continue and the strength of the mixes can increase.
- On the other hand, the evolution of pH and UCS in the LFS-stabilized mixes, shown in Fig. 12b, was very different. In this case, although the initial pH values (at 0 days) were similar to those obtained with lime, the decrease in pH over time was much lower. There was an extremely slight downward trend in the three mixes, presenting a slope of 1.8% for SAE5, 1.7% for SBE5, and 2.2% for SVE5. The highly alkaline initial environment was maintained over time with values above 11.2, which suggests the notable presence of calcium and magnesium oxides that favor the initiation and the development of pozzolanic reactions that improve strength. Strength improvement was especially noteworthy in the SBE5 mix (1612 kPa at 90 days). The soil stabilization experiences of Manso et al. (2013) with a similar LFS gave rise to a more pronounced decrease in pH over the 54 days of the control period. The interactions between soil and LFS were more complex than between soil and lime and the pozzolanic reactions involved the binding of calcium oxide and magnesium oxide (Manso et al. 2013), so a continuous increase of strength in all the LFS-stabilized mixes over time was noted.

### X-ray diffraction (XRD)

The changes caused by the LFS in the geotechnical properties of the soils can be explained by referring to the microstructural analysis of the LFS-stabilized soils. As with the untreated soils (the “Natural soils” section), XRD and SEM analyses were performed on the different mixtures stabilized with LFS.

**Fig. 13:**
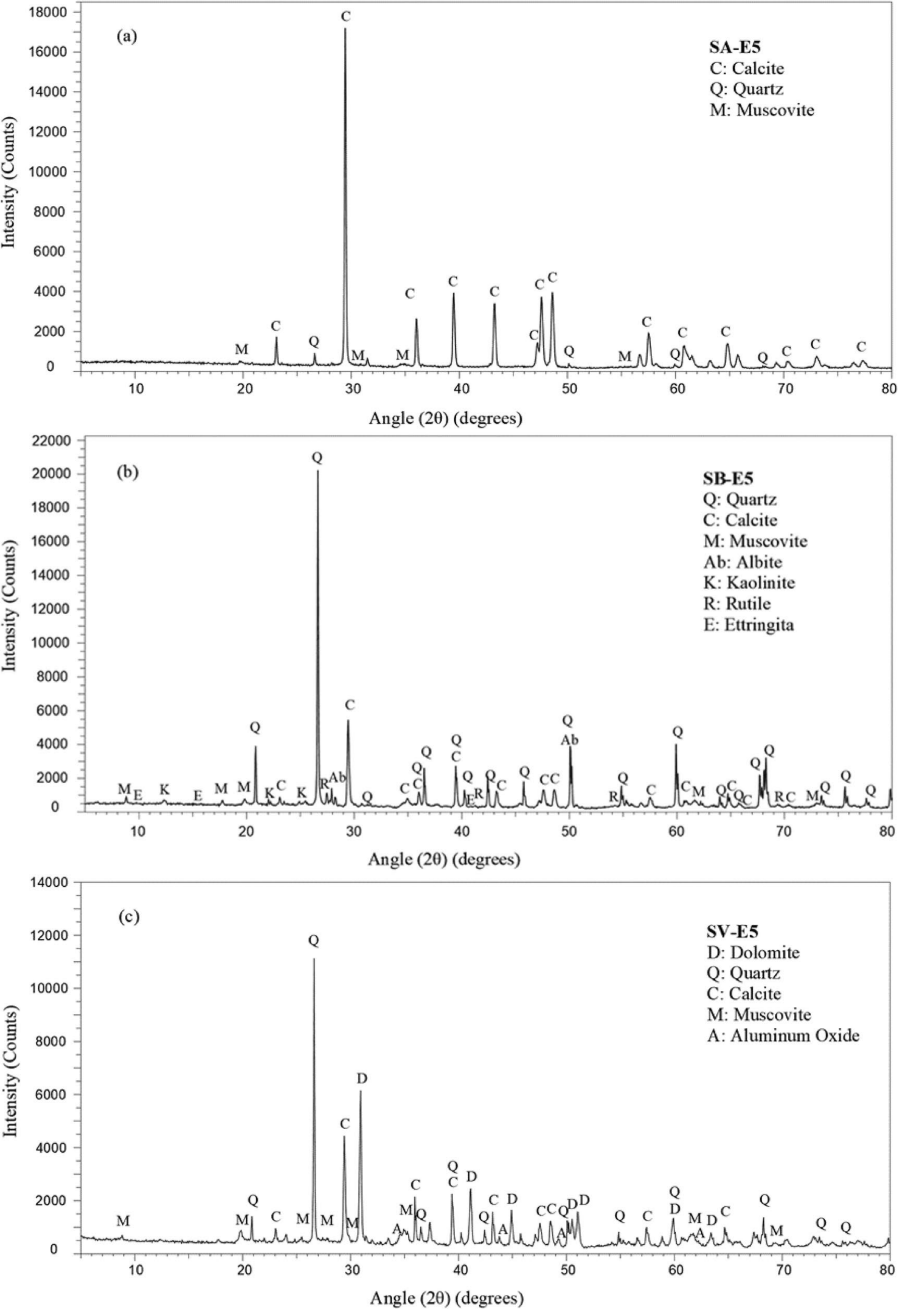
XRD patterns of LFS-stabilized soils at 90 days of curing time: **a** SAE5; **b** SBE5; **c** SVE5

XRD was used to identify the mineralogical components present in the samples when LFS was added to the clay materials, which depended on the intensity of the reactions that occurred during the different stabilization processes. Figure 13 shows the XRD patterns of the three soils mixed with 5% LFS after 90 days of curing to prompt those reactions. The analysis of the results is presented alongside the images of the untreated soils (Fig. 1).

The samples of the three soil mixes have practically the same mineralogical composition as the untreated soils. Nevertheless, the relative decrease of the quartz peak intensity is remarkable in all the mixes, which indicated that quartz was gradually consumed to form other compounds during the curing process, such as silica gels, responsible for the strength improvement of the stabilized soils. For instance, at 2*θ* value of 26.63° (3.34 Å), the quartz peak intensity decreased from the values of the untreated soils (1065 cps in SA, 20,301 cps in SB and 11,655 cps in SV, Fig. 1) to 543 cps in SAE5, 19,304 cps in SBE5, and 10,481 cps in SVE5 (Fig. 13). With respect to the calcite peaks of the SAE5 and SVE5 mixes, there was a decrease in the intensity of the different positions in which it was detected.

The most significant changes occurred in the SBE5 mixture. Vermiculite with very low presence in the soil sample is not detected in this sample, and the concentration of kaolinite became minor. In the positions where illite was detected in the soil sample SB at 2*θ* value of 8.872° (9.959 Å), 17.789° (4.982 Å), 25.516° (3.488 Å), and 45.416° (1.995 Å), in the mixture SBE5, muscovite was detected, as both of them have very similar structure and compositions. Therefore, the intensity of the calcite peak intensity increased, i.e., at 2*θ* about 29.45° (3.03 Å), which was attributed to calcite and CSH (Wang et al. 1995). There was also an increase in peak intensity from 3570 cps in the untreated soil to 5001 cps in the LFS-stabilized soil, which pointed to the formation of CSH gels. In addition, a higher percentage of albite and traces of ettringite were also detected, which indicated that the pozzolanic reactions were taking place. All these changes were in accordance with the strength improvements found in this soil sample in the UCS tests.

### Scanning electron microscopy (SEM)

**Fig. 14:**
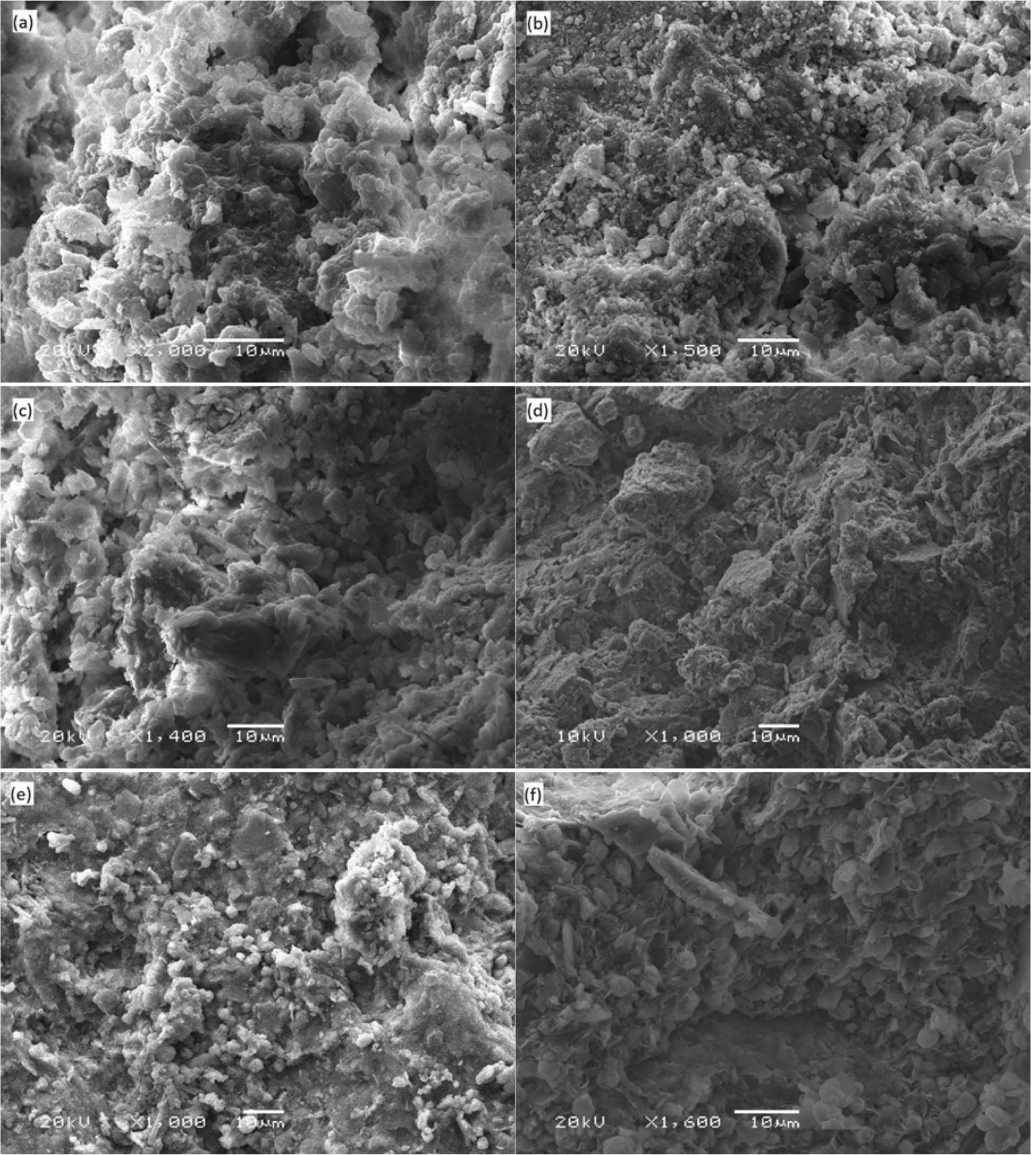
SEM images of mixes **a** SAC2; **b** SAE5; **c** SBC2; **d** SBE5; **e** SVC2; **f** SVE5 (scale bar: 10 μm)

With the aim of both evaluating the interaction of the clay particles with the stabilizers and verifying the formation of cementitious components, natural soils and the soil mixes stabilized with binders (2% lime or 5% LFS) were also studied by SEM analysis after a curing period of 90 days. Figure 14 shows the SEM images of the samples that were analyzed: a and b, for the SA mixes; c and d, for the SB mixes; and, e and f, for the SV mixes.

The type of clay (kaolinite, illite, montmorillonite…) clearly influences the reactions that can occur during the stabilization treatment when treated with lime (Eades and Grim 1960). In kaolinite, there is only a surface coating of the particles of a new phase where lime is present in kaolinite, while replacement of the exchange cations for the calcium within the lime occurs in montmorillonite and, in general, in other clays within the smectite group. The products resulting from the hydration of the components cover the surface of the soil particles, strengthen the bonds between them, and fill the pores of the amended soils, yielding more compact structures and, ultimately, improving mechanical strength (CBR, UCS) (Wu et al. 2019; Hossein Rafiean et al. 2020). The intensity and number of bonds that are formed will also depend on curing time and temperature. These microstructural changes within the stabilized soils are invariably in agreement with the results of the mechanical tests (Lopes et al. 2022).

The agglomeration of particles and a slight development of pozzolanic compacted minerals are visible in the SAE5 image, a fact related with the good UCS for this mix (1095 kPa). The image of mix SAC2 shows clustered agglomerated microstructures, although there was no increase in UCS in this mix (686 kPa), which was very similar to the UCS of the untreated soil. This observation indicated a dearth of pozzolanic reactions, confirmed by the low pH levels, shown in the “Volumetric stability tests” section, suggesting that the percentage of lime was insufficient to achieve the chemical reactions required for stabilization to take place (Eades and Grim 1966).

The SEM image showed a relatively compact microstructure for the SBE5 mix with particle agglomerations, and with almost no voids between the newly formed materials. Moreover, XRD pattern of SBE5 mix (Fig. 13) suggested the presence of CSH that confirms a behavior in accordance with its high UCS (1612 kPa) after 90 days of curing. The SBC2 mix showed a similar micro-structure to the SBE5 mix, although its strength at 90 days (944 kPa) was slightly lower than that of the SBE5 mix. Furthermore, this strength was maintained constant from the third day of curing. These constant UCS values at early curing ages confirmed that the development of strength depended on the amount of free lime available (Eades et al. 1962), which greatly decreased with curing time, as the pH values confirmed.

In the SVE5 image, some acicular elements associated with the growth of pozzolanic bonds can be observed. The micro-structure shows evidence of a flocculated structure and the formation of cementitious products, probably CSH, but XRD pattern (Fig. 13) did not verify this. Based on the evolution of its strength over time (1114 kPa after 90 days of curing), most of the cementitious chemical reactions had already occurred when this image was taken, as no large increases were experienced at 28 days of curing. The SVC2 mix also showed particle agglomerations, with some macro-elements, but without clearly distinguishable pozzolanic bonds, related with its positive improvement in UCS (840 kPa), albeit slightly lower than in the SVE5 mix.

## Conclusions

The application of LFS for stabilizing low-bearing-capacity soils can be regarded as a sustainable solution to enhance their engineering properties. This approach accomplishes a dual objective. Firstly, reuse this industrial by-product, as LFS use in soil stabilization reduces the environmental impact by minimizing the volume of this waste destined to landfill. Secondly, the substitution of conventional binders (such as lime or cement) presents a promising solution to decrease CO_2_ emissions associated with their production. Nevertheless, the technical viability of this solution has to be evaluated, aspects that in addressed in this paper through an experimental campaign. Based on the results collected in this research, the following points can be concluded:

- The addition of LFS to the clay soils resulted, in general, in an increase of the LL and the PL, and, therefore, in a reduction of the PI, which meant that the mixes showed better in situ workability. Furthermore, the stabilizer binders reduced the Maximum Dry Density and increased the Optimum Moisture Content, determined through the Modified Proctor test.
- After a curing period of 90 days, the soil+5%-LFS mixes achieved greater UCS than the soil+2%-lime mixes. The highest UCS values were obtained in the soil with the highest silica content (69.06%), in which UCS three times higher than the value of the natural soil was reached. More strongly pozzolanic reactions could be expected because of the high silica content.
- The bearing capacity of the soil+5%-LFS mixes according to the CBR index underwent increases of 8 and 14 times higher than those of the natural soils. However, they were slightly lower than those of the soil+2%-lime mixes.
- The application of lime to reduce free swelling in these clayey soils proved more effective than the use of LFS. Moreover, short curing times significantly promoted stabilization reactions and led to reduced free swelling.
- Shear strength parameters, cohesion, and friction angle showed the best performance for soil+5%-LFS mixes than for soil+2%-lime mixes at day 28 of curing. When LFS was used as a stabilizing agent, cohesion gradually increased over time to reach a high value after 28 days. However, the internal friction angle in comparison with the original soil only acquired a slightly higher value.
- The study of pH evolution over the curing time confirmed that the basic environment generated by the binders, both with lime and LFS, favored the development of pozzolanic reactions. It resulted in adequate development of UCS.
- The mineralogical (XRD) and microstructural (SEM) characterization of the LFS-stabilized mixes after 90 curing days confirmed the consumption of silica and the presence of compounds resulting from cementitious processes, such as calcium silicate hydrate gels. Both aspects were related with the physical and mechanical soil improvements, such as void reduction, more compact structure, and good strength.

In summary, the use of LFS as a stabilizer for low-bearing-capacity soils achieved a significant improvement in UCS, especially in the medium and long term, surpassing the results obtained with lime. Both binders contributed to reducing plasticity and significantly enhancing cohesion, which became more pronounced with increased curing time. The CBR index showed considerable improvement with both materials, although lime provided remarkable values. The lime treatment presented a better performance in reducing free swelling, but the importance of curing time was confirmed for both binders. Considering all these factors, LFS is a feasible technical solution for stabilizing clayey soils.

However, certain limitations were encountered in this study due to the fact that all the tests were performed with a single LFS content and type. In a similar way, a more extensive study of the evolution of cohesion with a longer curing time and with different percentages of LFS would provide a more rigorous knowledge of this feature. Future lines of research could therefore focus on evaluating the effect of different percentages of LFS, thus investigating its implications in the improvement of strength and reduction of plasticity. In addition, it would be a great challenge to analyze the importance of soil and LFS compositions in the performance of stabilization process, varying the soil typologies and introducing slags from different origins. The objective would be to propose effective working formulas for potential applications of LFS for in situ soil stabilization. Currently, the feasibility of incorporating other types of waste into soil stabilization is under investigation, exploring the potential inclusion of various industrial wastes in the process.

## Data Availability

The dataset presented in this study are available on request from the corresponding author.

## References

[CR1] Abdelkader MO, Hamdani SK (1985). Lime stabilisation for low cost roads in Egypt. Aust Road Res.

[CR2] Adolfsson D, Engström F, Robinson R, Björkman B (2011). Cementitious phases in ladle slag. Steel Res Int.

[CR3] Akin Altun I, Yilmaz I (2002). Study on steel furnace slags with high MgO as additive in Portland cement. Cem Concr Res.

[CR4] Akinmusuru JO (1991). Potential beneficial uses of steel slag wastes for civil engineering purposes. Resour Conserv Recycl.

[CR5] Akinwumi I (2014). Soil modification by the application of steel slag. Periodica Polytechnica Civil Eng.

[CR6] Anastasiou EK, Papayianni I, Papachristoforou M (2014). Behavior of self compacting concrete containing ladle furnace slag and steel fiber reinforcement. Mater Des.

[CR7] ASTM D1557-12 (2012). Standard test methods for laboratory compaction characteristics of soil using modified effort.

[CR8] ASTM D1883-21 (2021). Standard test method for California Bearing Ratio (CBR) of laboratory-compacted soils.

[CR9] ASTM D4546-21 (2021). Standard test methods for one-dimensional swell or collapse of soils.

[CR10] Behnood A (2018). Soil and clay stabilization with calcium- and non-calcium-based additives: a state-of-the-art review of challenges, approaches and techniques. Transp Geotech.

[CR11] Belhadj E, Diliberto C, Lecomte A (2012). Characterization and activation of basic oxygen furnace slag. Cem Concr Compos.

[CR12] Bell FG (1988). Stabilisation and treatment of clay soils with lime: part 1- basic principles. Ground Eng.

[CR13] Bell FG (1996). Lime stabilization of clay minerals and soils. Eng Geol.

[CR14] Bell FG, Coulthard JM (1990). Stabilization of clay soils with lime. Municipal Eng (Inst Civ Eng).

[CR15] Boardman DI, Glendinning S, Rogers CDF (2001). Development of stabilisation and solidification in lime-clay mixes. Geotechnique.

[CR16] Brand AS, Fanijo EO (2020). A review of the influence of steel furnace slag type on the properties of cementitious composites. Appl Sci.

[CR17] Brand AS, Singhvi P, Fanijo EO, Tutumluer E (2020). Stabilization of a clayey soil with ladle metallurgy furnace slag fines. Materials.

[CR18] CEDEX (2013) Escorias de acería de horno eléctrico. In: Ficha técnica. Catálogo de residuos utilizables en construcciónCentro de Estudios y Experimentación de Obras Públicas(CEDEX). Ministerio de Fomento, Madrid. Spain

[CR19] Consoli NC, Foppa D, Festugato L, Heineck KS (2007). Key parameters for strength control of artificially cemented soils. J Geotech Geoenviron.

[CR20] Coutinho A, Gonçalves A (1988). Production and properties of concrete.

[CR21] Das BM (1999) Principles of foundation engineeringPWS Publishing, Pacific Grove

[CR22] Dash SK, Hussain M (2012). Lime stabilization of soils: reappraisal. J Mater Civ Eng.

[CR23] Diniz DH, de Carvalho JMF, Mendes JC, Peixoto RAF (2017). Blast oxygen furnace slag as chemical soil stabilizer for use in roads. J Mater Civ Eng.

[CR24] Eades JL, Grim RE (1960). Reaction of hydrated lime with pure clay minerals in soil stabilization. Highway Res Board Bullet.

[CR25] Eades JL, Grim RE (1966). A quick test to determine lime requirements for lime stabilization. Highw Res Rec.

[CR26] Eades JL, Nichols F, Grim RE (1962). Formation of new minerals with lime stabilization as proven by field experiments in Virginia. Highw Res Board Bull.

[CR27] Euronorm EN (n.d.) European Committee for Standardization. Rue de Stassart, 36 Belgium–1050 Brussels

[CR28] Geiseler J (1996). Use of steelworks slag in Europe. Waste Manag.

[CR29] Ghobadi MH, Abdilor Y, Babazadeh R (2014). Stabilization of clay soils using lime and effect of pH variations on shear strength parameters. Bull Eng Geol Environ.

[CR30] Goodarzi AR, Salimi M (2015). Stabilization treatment of a dispersive clayey soil using granulated blast furnace slag and basic oxygen furnace slag. Appl Clay Sci.

[CR31] Herrmann I, Andreas L, Diener S, Lind L (2010). Steel slag used in landfill cover liners: laboratory and field tests. Waste Manag Res.

[CR32] Hossein Rafiean A, Najafi Kani E, Haddad A (2020). Mechanical and durability properties of poorly graded sandy soil stabilized with activated slag. J Mater Civ Eng.

[CR33] Ikeagwuani CC, Nwonu DC (2019). Emerging trends in expansive soil stabilisation: a review. J Rock Mech Geotech Eng.

[CR34] ISO 17892-12 (2012). Geotechnical investigation and testing. Laboratory testing of soil. Part 12: determination of liquid and plastic limits. European Committee for Standardization.

[CR35] ISO 17892-7 (2017). Geotechnical investigation and testing. Laboratory testing of soil. Part 7: unconfined compression test. European Committee for Standardization.

[CR36] ISO 17892-10 (2018). Geotechnical investigation and testing. Laboratory testing of soil. Part 10: direct shear test. European Committee for Standardization.

[CR37] ISO 10390 (2021). 10390: Soil, treated biowaste and sludge. Determination of pH. European Committee for Standardization.

[CR38] James J, Pandian PK (2016). Industrial wastes as auxiliary additives to cement/lime stabilization of soils. Adv Civ Eng.

[CR39] Kambole C, Paige-Green P, Kupolati WK, Ndambuki JM, Adeboje AO (2017). Basic oxygen furnace slag for road pavements: a review of material characteristics and performance for effective utilisation in southern Africa. Constr Build Mater.

[CR40] Kanagawa A, Kuwayama T (1997). The improvement of soft clayey soil utilizing reducing slag produced from electric arc furnace. DENKI-SEIKO.

[CR41] Katz LE, Rauch AF, Liljestrand HM, Harmon JS, Shaw KS, Albers H (2001). Mechanisms of soil stabilization with liquid ionic stabilizer. Transp Res Record.

[CR42] Kim YS, Tran TQ, Kang GO, Do TM (2019). Stabilization of a residual granitic soil using various new green binders. Constr Build Mater.

[CR43] Lopes EC, da Silva TO, Pitanga HN, Pedroti LG, Franco de Carvalho JM, Nalon GH, de Lima GES, de Araújo END (2022) Stabilisation of clayey and sandy soils with ladle furnace slag fines for road construction. Road Mater Pavement Design. 10.1080/14680629.2021.2017328

[CR44] Maghool F, Arulrajah A, Du YJ, Horpibulsuk S, Chinkulkijniwat A (2017). Environmental impacts of utilizing waste steel slag aggregates as recycled road construction materials. Clean Techn Environ Policy.

[CR45] Maghool F, Arulrajah A, Horpibulsuk S, Du YJ (2017). Laboratory evaluation of ladle furnace slag in unbound pavement-base/subbase applications. J Mater Civ Eng.

[CR46] Mahesh Bhat K, Nayak S (2021). Experimental studies and its application using PLAXIS-2D for lithomargic clay stabilized by GBFS and lime. Geotech Geol Eng.

[CR47] Mahieux PY, Aubert JE, Escadeillas G (2009). Utilization of weathered basic oxygen furnace slag in the production of hydraulic road binders. Constr Build Mater.

[CR48] Mahoutian M, Shao Y (2016). Low temperature synthesis of cement from ladle slag and fly ash. J Sustain Cement-Based Mater.

[CR49] Malhotra VM, Mehta PK (2004). Pozzolanic and cementitious materials.

[CR50] Manh Do T, Kang G-O, Kim Y-s (2019). Development of a new cementless binder for controlled low strength material (CLSM) using entirely by-products. Constr Build Mater.

[CR51] Manso JM, Losañez M, Polanco JA, Gonzalez JJ (2005). Ladle furnace slag in construction. J Mater Civ Eng.

[CR52] Manso JM, Ortega-López V, Polanco JA, Setién J (2013). The use of ladle furnace slag in soil stabilization. Constr Build Mater.

[CR53] Manso JM, Rodriguez A, Aragón A, Gonzalez JJ (2011). The durability of masonry mortars made with ladle furnace slag. Constr Build Mater.

[CR54] Marinho ALB, Mol Santos CM, de Carvalho JMF, Mendes JC, Brigolini GJ, Peixoto RAF (2017). Ladle furnace slag as binder for cement-based composites. J Mater Civ Eng.

[CR55] Ministerio de Fomento (2002). Pliego de Prescripciones Técnicas Generales para obras de carreteras y puentes.

[CR56] Ministerio de Fomento (2015). Pliego de prescripciones técnicas generales para obras de carreteras y puentes. Artículo 512: Suelos estabilizados in situ Madrid. Orden FOM/2523/2014.

[CR57] Mitchell JK, Hooper DR (1961). Influence of time between mixing and compaction on properties of lime stabilized expansive clay. Highway Res Board Bullet.

[CR58] Montenegro-Cooper JM, Celemín-Matachana M, Cañizal J, González JJ (2019). Study of the expansive behavior of ladle furnace slag and its mixture with low quality natural soils. Constr Build Mater.

[CR59] Montenegro JM, Celemín-Matachana M, Cañizal J, Setién J (2013). Ladle furnace slag in the construction of embankments: expansive behavior. J Mater Civ Eng.

[CR60] Mosa AM, Taher AH, Al-Jaberi LA (2017). Improvement of poor subgrade soils using cement kiln dust. Case Stud Constr Mater.

[CR61] Motz H, Geiseler J (2001). Products of steel slags an opportunity to save natural resources. Waste Manag.

[CR62] Nidzam RM, Kinuthia JM (2010). Sustainable soil stabilisation with blastfurnace slag - a review. Proceed Inst Civ Eng Constr Mater.

[CR63] Obuzor GN, Kinuthia JM, Robinson RB (2012). A mitigation to flooding effects on road structural layers/embankments constructed on floodplains. Eng Geol.

[CR64] Ortega-López V, Manso JM, Cuesta II, González JJ (2014). The long-term accelerated expansion of various ladle-furnace basic slags and their soil-stabilization applications. Constr Build Mater.

[CR65] Ortega-López V, Skaf M, Santamaría A (2017). The reuse of ladle furnace basic slags in clayey soil-stabilization applications. Soil Stabilization: Types, Methods and Applications.

[CR66] Papayianni I, Anastasiou E (2006) Optimization of ladle furnace slag for use as a supplementary cementing material. Measuring, Monitoring and Modeling Concrete Properties. Konsta-Gdoutos MS (eds), Springer, Dordrecht, pp 411–417. 10.1007/978-1-4020-5104-3_50

[CR67] Papayianni I, Anastasiou E (2010). Production of high-strength concrete using high volume of industrial by-products. Constr Build Mater.

[CR68] Papayianni I, Anastasiou E (2012). Effect of granulometry on cementitious properties of ladle furnace slag. Cem Concr Compos.

[CR69] Petry TM, Little DN (2002). Review of stabilization of clays and expansive soils in pavements and lightly loaded structures - history, practice, and future. J Mater Civ Eng.

[CR70] Poh HY, Ghataora GS, Ghazireh N (2006). Soil stabilization using basic oxygen steel slag fines. J Mater Civ Eng.

[CR71] Polanco JA, Manso JM, Setién J, González JJ (2011). Strength and durability of concrete made with electric steelmaking slag. ACI Mater J.

[CR72] Proctor DM, Fehling KA, Shay EC, Wittenborn JL, Green JJ, Avent C, Bigham RD, Connolly M, Lee B, Zak MA, Shepker TO (2000). Physical and chemical characteristics of blast furnace, basic oxygen furnace, and electric arc furnace steel industry slags. Environ Sci Technol.

[CR73] Radenović A, Malina J, Sofilić T (2013). Characterization of ladle furnace slag from carbon steel production as a potential adsorbent. Adv Mater Sci Eng.

[CR74] Rahmat MN, Kinuthia JM (2011). Compaction of fills involving stabilisation of expansive soils. Proceed Inst Civ Eng Geotechn Eng.

[CR75] Richardson IG, Cabrera JG (2000). Nature of C-S-H in model slag-cements. Cem Concr Compos.

[CR76] Rodriguez A, Manso JM, Aragon A, Gonzalez JJ (2009). Strength and workability of masonry mortars manufactured with ladle furnace slag. Res Conserv Recycling.

[CR77] Rogers CDF, Glendinning S (2000). Lime requirement for stabilization. Transp Res Record.

[CR78] Rosales J, Cabrera M, Agrela F (2017). Effect of stainless steel slag waste as a replacement for cement in mortars. Mechanical and statistical study. Constr Build Mater.

[CR79] Santamaria A, Faleschini F, Giacomello G, Brunelli K, San José J-T, Pellegrino C, Pasetto M (2018). Dimensional stability of electric arc furnace slag in civil engineering applications. J Clean Prod.

[CR80] Santamaría A, Ortega-López V, Skaf M, Faleschini F, Orbe A, San-José JT, de Brito J, Thomas C, Medina C, Agrela F (2021). Ladle furnace slags for construction and civil works: A promising reality. Waste and byproducts in cement-based materials.

[CR81] Santamaría A, Rojí E, Skaf M, Marcos I, González JJ (2016). The use of steelmaking slags and fly ash in structural mortars. Constr Build Mater.

[CR82] Seco A, Del Castillo JM, Espuelas S, Marcelino-Sadaba S, Garcia B (2021). Stabilization of a clay soil using cementing material from spent refractories and ground-granulated blast furnace slag. Sustain (Switzerland).

[CR83] Serjun VZ, Mirtič B, Mladenovič A (2013). Evaluation of ladle slag as a potential material for building and civil engineering. Materiali in Tehnologije.

[CR84] Setién J, Hernández D, González JJ (2009). Characterization of ladle furnace basic slag for use as a construction material. Constr Build Mater.

[CR85] Shen J, Xu Y, Chen J, Wang Y (2019). Study on the stabilization of a new type of waste solidifying agent for soft soil. Materials.

[CR86] Sherwood P (1993). Soil stabilization with cement and lime: state-of-the-art review.

[CR87] Shi C (2002). Characteristics and cementitious properties of ladle slag fines from steel production. Cem Concr Res.

[CR88] Shi C (2004). Steel slag - its production, processing, characteristics, and cementitious properties. J Mater Civ Eng.

[CR89] Shi C, Day RL (1999). Early strength development and hydration of alkali-activated blast furnace slag/fly ash blends. Adv Cem Res.

[CR90] Shi C, Qian J (2000). High performance cementing materials from industrial slags — a review. Resour Conserv Recycl.

[CR91] Taylor HF (1997). Cement chemistry.2.

[CR92] Thompson MR (1969). Engineering properties of lime-soil mixtures. J Mater.

[CR93] USGS (2023) Iron and steel slag. https://www.usgs.gov/centers/national-minerals-information-center/iron-and-steel-slag-statistics-and-information. Accessed 25 May 2023

[CR94] Wang G, Wang Y, Gao Z (2010). Use of steel slag as a granular material: Volume expansion prediction and usability criteria. J Hazard Mater.

[CR95] Wang SD, Pu XC, Scrivener KL, Pratt PL (1995). Alkali-activated slag cement and concrete: a review of properties and problems. Adv Cem Res.

[CR96] Wild S, Kinuthia JM, Jones GI, Higgins DD (1998). Effects of partial substitution of lime with ground granulated blast furnace slag (GGBS) on the strength properties of lime-stabilised sulphate-bearing clay soils. Eng Geol.

[CR97] Wilkinson A, Haque A, Kodikara J (2010). Stabilisation of clayey soils with industrial by-products: part A. Proceed Inst Civ Eng-Ground Improve.

[CR98] Wilkinson A, Haque A, Kodikara J (2010). Stabilisation of clayey soils with industrial by-products: part B. Proceed Inst Civ Eng-Ground Improve.

[CR99] World Steel Association (2023) World press release. https://worldsteel.org. Accessed 25 May 2023

[CR100] Wu J, Liu Q, Deng Y, Yu X, Feng Q, Yan C (2019). Expansive soil modified by waste steel slag and its application in subbase layer of highways. Soils Found.

[CR101] Xu B, Yi Y (2019). Soft clay stabilization using ladle slag-ground granulated blastfurnace slag blend. Appl Clay Sci.

[CR102] Yildirim IZ, Prezzi M (2011). Chemical, mineralogical, and morphological properties of steel slag. Adv Civ Eng.

[CR103] Yildirim IZ, Prezzi M (2017). Experimental evaluation of EAF ladle steel slag as a geo-fill material: mineralogical, physical & mechanical properties. Constr Build Mater.

[CR104] Yoon S, Balunaini U, Yildirim IZ, Prezzi M, Siddiki NZ (2009). Construction of an embankment with a fly and bottom ash mixture: field performance study. J Mater Civ Eng.

